# Electron Dynamics in Alkane C–H Activation Mediated by Transition Metal Complexes

**DOI:** 10.1021/acs.jpca.4c01131

**Published:** 2024-06-04

**Authors:** Yu-Ho Cheng, Yeu-Shiuan Ho, Chia-Jung Yang, Chun-Yu Chen, Chi-Tien Hsieh, Mu-Jeng Cheng

**Affiliations:** Department of Chemistry, National Cheng Kung University, Tainan 701, Taiwan

## Abstract

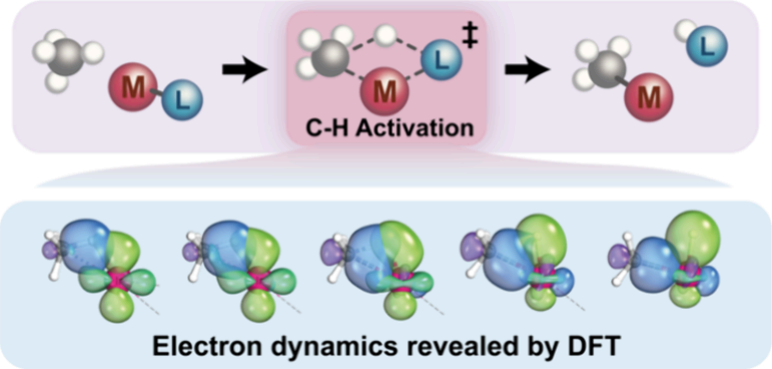

Alkanes, ideal raw materials for industrial chemical production, typically exhibit limited reactivity due to their robust and weakly polarized C–H bonds. The challenge lies in selectively activating these C–H bonds under mild conditions. To address this challenge, various C–H activation mechanisms have been developed. Yet, classifying these mechanisms depends on the overall stoichiometry, which can be ambiguous and sometimes problematic. In this study, we utilized density functional theory calculations combined with intrinsic bond orbital (IBO) analysis to examine electron flow in the four primary alkane C–H activation mechanisms: oxidative addition, σ-bond metathesis, 1,2-addition, and electrophilic activation. Methane was selected as the representative alkane molecule to undergo C–H heterolytic cleavage in these reactions. Across all mechanisms studied, we find that the CH_3_ moiety in methane consistently uses an electron pair from the cleaved C–H bond to form a σ-bond with the metal. Yet, the electron pair that accepts the proton differs with each mechanism: in oxidative addition, it is derived from the *d*-orbitals; in σ-bond metathesis, it resulted from the metal–ligand σ-bonds; in 1,2-addition, it arose from the π-orbital of the metal–ligand multiple bonds; and in electrophilic activation, it came from the lone pairs on ligands. This detailed analysis not only provides a clear visual understanding of these reactions but also showcases the ability of the IBO method to differentiate between mechanisms. The electron flow discerned from IBO analysis is further corroborated by results from absolutely localized molecular orbital energy decomposition analysis, which also helps to quantify the two predominant interactions in each process. Our findings offer profound insights into the electron dynamics at play in alkane C–H activation, enhancing our understanding of these critical reactions.

## 1 Introduction

Alkanes, major components of natural gas and petroleum, are the most cost-effective and abundant precursors for industrial chemical production. However, replacing a hydrogen atom in a carbon–hydrogen (C–H) bond with another element or functional group to form complex, value-added structures poses significant challenges. This difficulty arises from these bonds being thermodynamically strong and kinetically inactive. Consequently, alkanes are mainly used as fuels, where their bond energy is released as heat. Therefore, the efficient and selective activation of alkane C–H bonds under mild conditions represents a promising avenue with considerable economic implications.

Encouraged by the seminal work of Shilov and Shul'pin,^1^ a diverse array of methodologies has been developed for the activation of alkane C–H bonds via metal complexes, incorporating different mechanistic pathways. These methodologies have been comprehensively chronicled in a number of outstanding review articles.^2−6^ The recent review by Altus and Love, which is devoted to the discussion of transition metal-mediated C–H activation, organizes these mechanisms into four principal categories: (a) oxidative addition, (b) σ-bond metathesis, (c) 1,2-addition, and (d) electrophilic activation.^7^

**Scheme 1:**
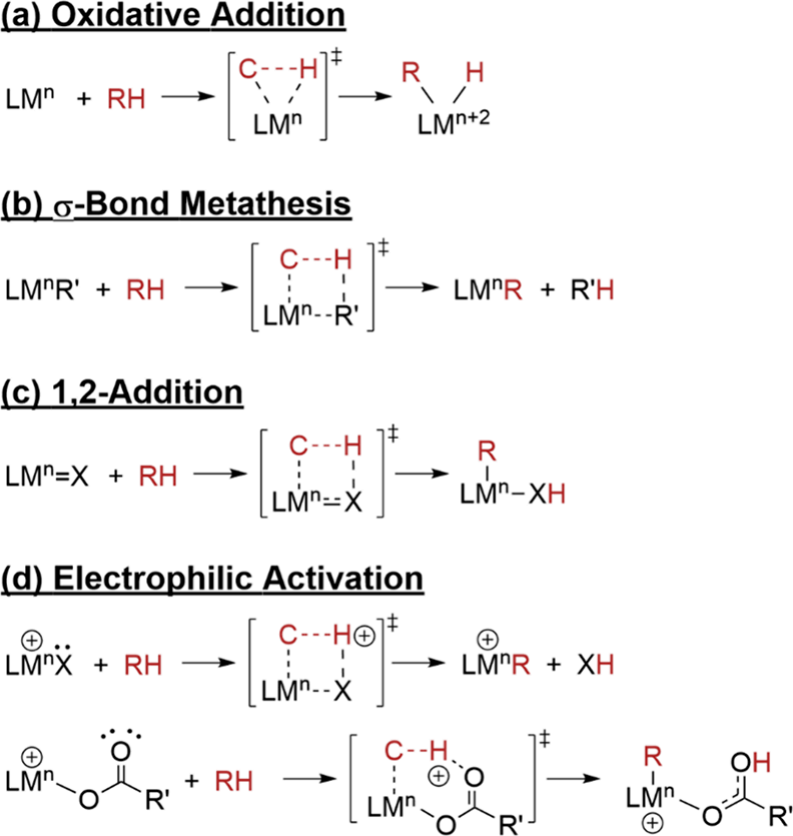
Schematic Description of Four C–H Activation Mechanisms

Oxidative addition typically occurs in low valent, electron-rich metal complexes with strongly donating ligands (Scheme 1 a). The reaction progresses through a three-membered ring transition state. In this process, the metal’s oxidation state and coordination number both increase by two. σ-Bond metathesis typically involves early transition metals that lack *d*-electrons available for oxidative addition (Scheme 1b). The process unfolds via a four-centered transition state, ultimately leading to the substitution of the M-R’ σ-bond with an M-R σ-bond. It is important to note that the metal retains its oxidation state throughout the course of the reaction.

1,2-Addition is generally associated with early transition metals. In this mechanism, C–H of alkane adds across an M–X double (or triple) bond to form M–C and X–H, as shown in Scheme 1c. Throughout this addition process, the oxidation state of the metal is preserved. Electrophilic activation, as shown in Scheme 1d, requires the coordination of an electropositive metal that withdraws electron density from C–H bonds. This process enhances the acidity of the hydrogen atom, facilitating its abstraction by a lone pair from an internal (or external) base. The reaction can proceed through a four- or six-centered transition state.

The classification of C–H activation mechanisms conventionally relies on the overall stoichiometry, but this approach can be ambiguous and sometimes problematic. For example, metal complexes possessing an M-X bond, where X is an atom with available lone pairs, may initiate C–H bond activation through either σ-bond metathesis or electrophilic activation pathways. Differentiating between these two mechanisms is challenging since they yield indistinguishable products. This dilemma is evidenced in the work of Periana and Goddard, who initially proposed that C–H cleavage by an Ir–OH complex occurred through σ-bond metathesis but found that the actual pathway followed electrophilic activation in later studies.^8,9^

Knizia et al. recently put forward an innovative approach that integrates DFT calculations with the intrinsic bond orbital (IBO) localization scheme to effectively track electron flow during chemical reactions.^10,11^ The method has been successfully applied to map out electron flow in various chemical reactions^12−15^ as well as uncover the nature of hydrogen transfer.^16−19^ Recently, we have used this approach to track electron flow in methane electrochemical oxidation to methanol using surface-bound oxygen on N-doped graphene.^20^ In the present research, we applied this methodology to examine representative cases of four C–H activation mechanisms. Our objective is to provide a visually clear approach for distinguishing among these mechanisms and understand the electron dynamics during the process.

## 2 Computational Details

All DFT calculations including geometry optimization and vibrational frequency calculations were carried out using Gaussian16 at the B3LYP-D3/def2-SVP level.^21−24^ Transition states were confirmed through frequency calculations, which exhibited a single imaginary frequency along the reaction coordinate. To achieve more accurate electronic energy, single-point calculations using the improved basis set, def2-TZVP, were performed on structures optimized at the B3LYP-D3/def2-SVP level. The principal discussion is based on energetics from B3LYP-D3/def2-SVP, while results derived from B3LYP-D3/def2-TZVP//B3LYP-D3/def2-SVP are included in the Supporting Information (SI) for reference.

Intrinsic reaction coordinate (IRC) calculations were performed for approximately 200 points along the reaction pathway at the B3LYP-D3/def2-SVP level of theory. Orbital localization via the IBO scheme necessitated single-point recalculations for each point along the IRC paths, which were executed with the ORCA package at the same level as the IRC calculations. The evolution of localized orbitals throughout the IRC was tracked using IboView.^25^ The energy decomposition analysis based on absolutely localized molecular orbitals (ALMO-EDA)^26−28^ was performed using the Q-Chem package at the B3LYP-D3/def2-SVP level.

## 3 Results and Discussion

The field of alkane C–H bond activation has seen extensive study for more than four decades.^7^ In our current work, we concentrate on specific examples that epitomize the four primary C–H activation mechanisms. We began each section below with a concise introduction for each mechanism and its early examples followed by a detailed discussion of our calculated energetics and how these compare with earlier theoretical studies. Lastly, we offer insights into electron movement through IBO analysis that provides the basis for an arrow-pushing description of each mechanism.

### 3.1 C–H Activation through Oxidative Addition

Oxidative addition is among the earliest mechanisms proposed for alkane C–H activation. One early experiment, conducted by Bergman et al., showed that upon irradiation, (Cp*)(PMe_3_)Ir(H)_2_ (Cp* = η^5^-C_5_Me_5_) reacts with alkane (R-H) to form (Cp*)(PMe_3_)Ir(R)(H).^29,30^ A subsequent investigation revealed that upon photolysis, (Cp*)(PMe_3_)Ir(H)_2_ undergoes reductive elimination, leading to the release of H_2_ and the formation of a reactive (Cp*)(PMe_3_)Ir species. This newly formed species then participates in insertion into alkane C–H bonds.^31^

Another early experiment, performed by Graham et al., demonstrated that an alkane undergoes oxidative addition with (Cp*)(CO)_2_Ir to form (Cp*)(CO)Ir(R)(H) at room temperature upon irradiation.^32,33^ This process is analogous to that of (Cp*)(PMe_3_)Ir(H)_2_, where photolysis induces the formation of a reactive 16-electron (Cp*)(CO)Ir species. This process occurs through dissociation of one CO ligand, enabling the species to subsequently engage in C–H bond insertion.^34^

**Scheme 2:**
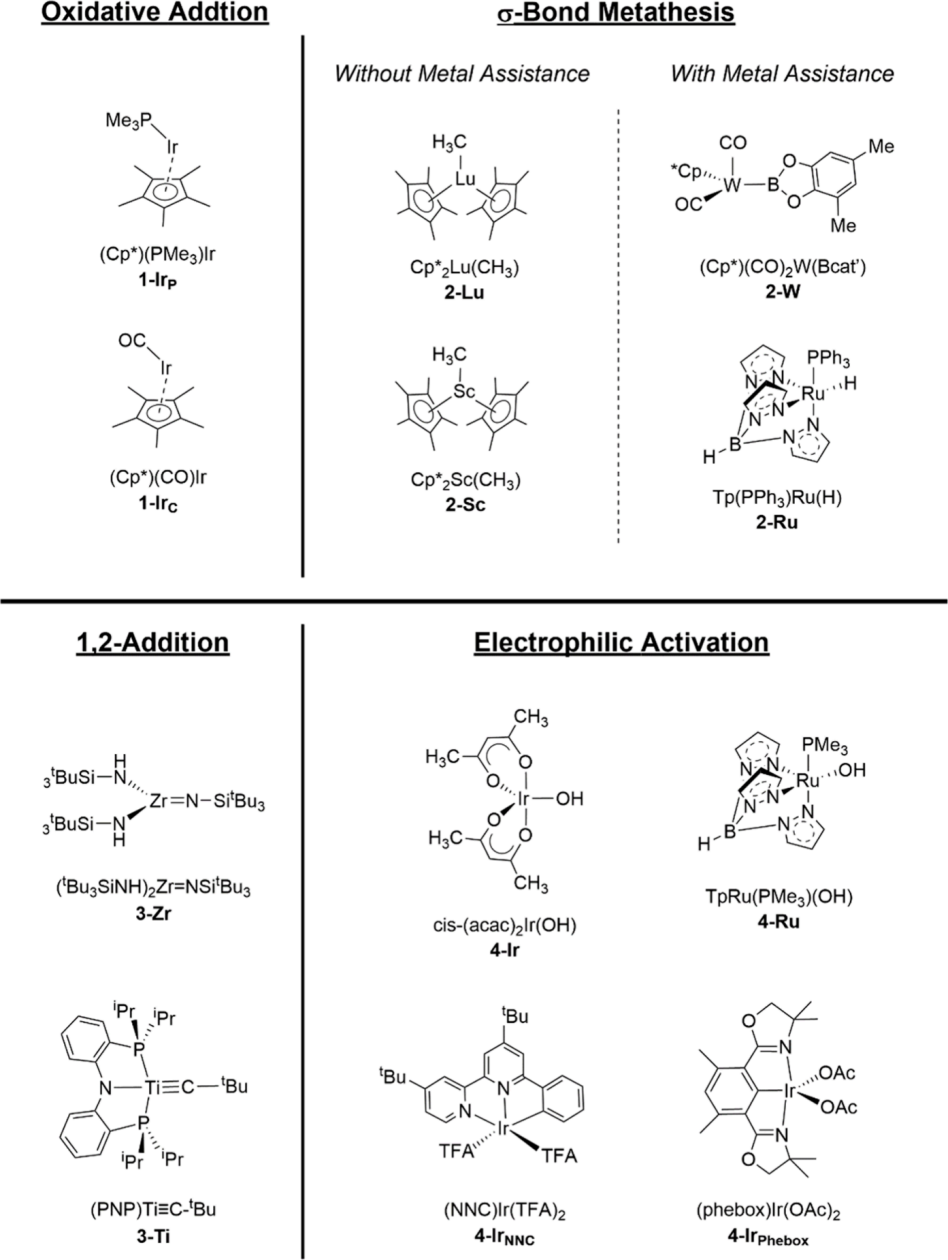
Two-Dimensional Description of the Metal Complexes Considered in This Work for Methane C–H Bond Activation through Four Distinct Mechanisms

In our study, methane was chosen as a representative alkane to determine the energetics of oxidative addition on (Cp*)(PMe_3_)Ir (**1-Ir**_**P**_, Scheme 2) and (Cp*)(CO)Ir (**1-Ir**_**C**_). We found that the ground states for both **1-Ir**_**P**_ and **1-Ir**_**C**_ are triplets, not singlets, with singlet–triplet energy gaps (Δ*E* = *E*_S_ – *E*_T_) of 3.6 and 1.0 kcal/mol, respectively, aligning with prior theoretical studies.^35,36^ However, it has been shown in a previous study that only the singlet state is capable of coordinating and reacting with methane.^37^ Therefore, our calculations of the energetics for methane’s oxidative addition were based on the singlet state.

We find that both **1-Ir**_**P**_ and **1-Ir**_**C**_ form a stable σ-bond complexes with methane with Δ*E* = −13.1 and −13.7 kcal/mol, respectively (Table 1). Subsequently, Ir inserts into the C–H bond of methane via a three-centered transition state. The energy barrier (Δ*E*^‡^) and reaction energy (Δ*E*) for this process are 0.10 and −28.8 kcal/mol, respectively, for **1-Ir**_**P**_ and 1.3 and −21.0 kcal/mol, respectively, for **1-Ir**_**C**_. The negligible barrier and substantial exothermicity suggest that the reaction is both kinetically facile and thermodynamically favorable. This outcome aligns with prior experimental studies by Graham et al. and Rest et al., which demonstrated that the reaction between methane and **1-Ir**_**C**_ can occur even at 12 K.^38,39^ In addition, our result is consistent with an earlier theoretical study by Ziegler et al., revealing that methane C–H activation by (Cp)(CO)Ir (where Cp = η^5^-C_5_H_5_) has a low Δ*E*^‡^ of only 2.4 kcal/mol.^40^

**Table 1:** Energetics of Methane C–H Activation across Different Metal Complexes Computed Using the B3LYP-D3/def2-SVP Level of Theory^a^

metal complex	**methane complex**	**TS**	**product**
oxidative addition			
(Cp*)(PMe_3_)Ir (**1-Ir**_**P**_)	–13.1	–13.0 (Δ*E*^‡^ = 0.1)	–41.9
(Cp*)(CO)Ir (**1-Ir**_**C**_)	–13.7	–12.4 (Δ*E*^‡^ = 1.3)	–34.7
σ-bond metathesis			
(Cp*)_2_Lu(CH_3_) (**2-Lu**)	–6.5	12.2 (Δ*E*^‡^ = 18.7)	–6.5
(Cp*)_2_Sc(CH_3_) (**2-Sc**)	–4.0	13.2 (Δ*E*^‡^ = 17.2)	–4.0
(Cp*)(CO)_2_W(Bcat’) (**2-W**)	–11.2	–0.6 (Δ*E*^‡^ = 10.6)	–10.7
(Tp)(PPh_3_)Ru(H) (**2-Ru**)	–9.3	0.5 (Δ*E*^‡^ = 9.8)	–4.5
1,2-addition			
(*t*-Bu_3_SiNH)_2_Zr = NSi-*t*-Bu_3_ (**3-Zr**)	–11.1	5.7 (Δ*E*^‡^ = 16.8)	–27.8
(PNP)Ti≡C-tBu (**3-Ti**)	–12.4	–6.7 (Δ*E*^‡^ = 5.7)	–40.0
electrophilic activation			
cis-(acac)_2_Ir(OH) (**4-Ir**)	–2.7	7.2 (Δ*E*^‡^ = 9.9)	–20.6
TpRu(PMe_3_)(OH) (**4-Ru**)	–5.0	10.4 (Δ*E*^‡^ = 15.4)	–1.9
(NNC)Ir(TFA)_2_(**4-Ir**_**NNC**_)	3.6	17.2 (Δ*E*^‡^ = 13.6)	6.5
(phebox)Ir(OAc)_2_(**4-Ir**_**Phebox**_)	13.4	20.7 (Δ*E*^‡^ = 7.3)	7.3

a All energetics are referenced to isolated metal complex and methane

**Figure 1:**
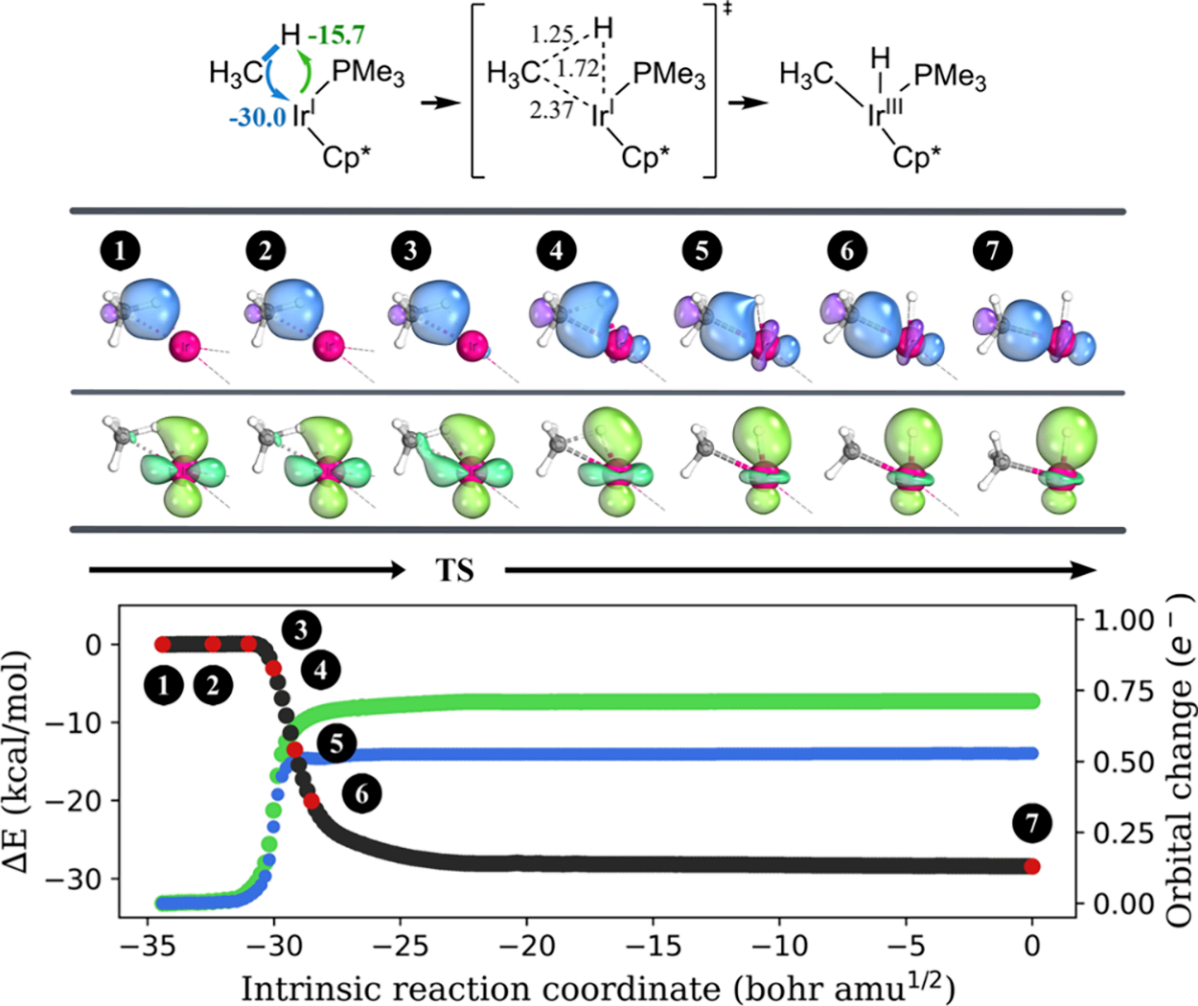
Progression of the IBO localized orbitals throughout methane oxidative addition on **1-Ir**_**P**_. Each curly arrow is accompanied by a numerical value (in kcal/mol) to indicate its contribution to charge transfer stabilization at the transition state structure, as computed by ALMO-EDA. The important bond lengths in the transition state structure are shown, and the unit is angstrom. For each orbital evolution, a consistent color was used across the three graphs in this figure. A similar analysis was obtained for **1-Ir**_**C**_ + CH_4_ and is summarized in Figure S1 in the SI. For each point *s* on the IRC and each IBO *i*, a charge vector q(*s*, *i*) is defined, with components ***q_A_***(*s*, *i*) representing the IAO partial charge of atom A. The orbital change is then calculated as , where *n* is the total number of atoms in the system and *s* = 0 is to indicate the initial point on the IRC (the reactant).

Next, we performed IBO analysis along the course of methane oxidative addition to **1-Ir**_**P**_ and **1-Ir**_**C**_. In both cases, the methyl group of methane forms a metal–carbon bond with Ir using the electron pair from the broken C–H bond (Figure 1, blue/purple color). Concurrently, Ir utilizes an electron pair from one of its *d*-orbitals to establish a metal–hydrogen bond with the transferring proton (lime/green color). Hence, Ir demonstrates ambiphilic characteristics by serving as an electrophile for the methyl anion and a nucleophile for the proton of methane. Our IBO analysis characterizes the oxidative addition mechanism as a formal [2σ + 2d] process. The arrow-pushing description of this C–H activation mechanism is also provided. The prior theoretical study by Vidossich et al., which examined the displacement of localized molecular orbital centroids during chemical reactions, proposed an arrow-pushing description for oxidative addition that aligns with our findings.^41^

### 3.2 C–H Activation through σ-Bond Metathesis

#### 3.2.1 σ-Bond Metathesis without Metal Assistance

σ-Bond metathesis is another early proposed mechanism for alkane C–H activation that typically involves *d*- and *f*-block metals with *d*^0^ configuration. Watson et al. and Bercaw et al. have significantly advanced this field.^42−44^ Watson et al. discovered that (Cp*)_2_MR’ (M = Lu, Y; R’ = CH_3_, H) complexes can cleave C–H bonds in various hydrocarbons (R-Hs, e.g., methane, benzene, and pyridine) to yield (Cp*)_2_MR and R’-H.^42,43^ Similarly, Bercaw et al.’s work showed that (Cp*)_2_Sc(CH_3_) reacts with a variety of R-Hs, leading to the formation of (Cp*)_2_Sc(R) and CH_4_.^44^

We calculated the energetics of methane C–H cleavage by (Cp*)_2_Lu(CH_3_) (**2-Lu**) and (Cp*)_2_Sc(CH_3_) (**2-Sc**). Our DFT calculations indicate that methane σ-bond complexes exhibit only marginally higher stability compared to their separated components (Δ*E* = −6.5 and −4.0 kcal/mol for **2-Lu** and **2-Sc**, respectively). This result is primarily due to the lack of back-donation from the metal to the vacant orbitals of methane.

Consistent with prior theoretical studies,^45,46^ our findings reveal a four-membered ring transition state composed of a M-CH_3_ group from the metal complex and a C–H bond from methane. For both systems, this four-membered ring features a nearly linear C–H–C moiety with angles of 179.2° for **2-Lu** and 177.8° for **2-Sc**, respectively. The Δ*E*^‡^s were computed as 18.7 kcal/mol for **2-Lu** and 17.2 kcal/mol for **2-Sc**, aligning well with the findings of ∼20.0 kcal/mol from a previous study by Eisenstein et al.^46^

**Figure 2:**
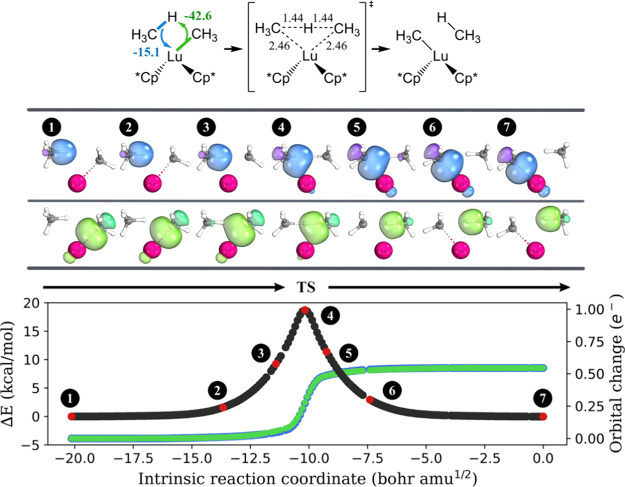
Progression of the IBO localized orbitals throughout the activation of the methane C–H bond via σ-bond metathesis by **2-Lu**. Each curly arrow is accompanied by a numerical value (in kcal/mol) to indicate its contribution to charge transfer stabilization at the transition state structure, as computed by ALMO-EDA. The important bond lengths in the transition state structure are shown, and the unit is angstrom. For each orbital evolution, a consistent color was used across the three graphs in this figure. A similar analysis was obtained for **2-Sc** + CH_4_ and is summarized in Figure S2 in the SI.

We then conducted an IBO analysis along the path of methane C–H activation by **2-Lu** and **2-Sc**. This analysis revealed that the methyl group from methane forms a metal–carbon σ-bond with the metal, similar to oxidative addition (Figure 2, blue/purple color). This bond is constructed using the electron pair from the cleaved C–H σ-bond. Unlike in oxidative addition, the electron pair in the M-CH_3_ σ-bond rather than the lone pair on the *d*-orbitals is utilized to accommodate the transferring proton, thereby forming a new C–H σ-bond (lime/green color). As a result, the IBO analysis characterizes the σ-bond metathesis mechanism as a formal [2σ + 2σ] process.

The nature of σ-bond metathesis as elucidated by our IBO analysis aligns with findings from previous research. Watson et al. have demonstrated that the rate of σ-bond metathesis escalates as the electrophilicity of the metal center increases.^47^ Moreover, Bercaw et al. have observed that the reaction rate of σ-bond metathesis increases as the p*K*_a_ value of the C–H bonds decreases (more acidic).^44^

#### 3.2.2 σ-Bond Metathesis with Metal Assistance

Experiments have shown that metal complexes with *d*-orbital electrons can also activate alkane C–H bonds via σ-bond metathesis.^48−52^ In these systems, the metal center is crucial for facilitating proton transfer as it forms a transient M–H bond during the reaction. The metal is partially oxidized as it transitions from the reactant to the transition state but reverts to its original oxidation state as the product forms. This process is distinct from conventional σ-bond metathesis, where no partial M-H bond forms and the metal is not oxidized during the process.

An early example of this reaction type was reported by Hartwig and co-workers.^48,49^ They synthesized (Cp*)(CO)_3_W(Bcat’) and (Cp*)(CO)_2_Fe(Bcat’) (Bcat’ = 1,2-O_2_C_6_H_2_-3,5-(CH_3_)_2_) and discovered that upon irradiation, these two complexes can activate and functionalize alkane C–H bonds at the terminal position to form alkylboronate esters. The formal oxidation states of W and Fe are both +2 with W possessing four electrons and Fe having six electrons in their respective *d*-orbitals. Further mechanistic studies revealed that under photolysis, one CO ligand dissociates, generating active 16-electron intermediates that initiate C–H bond cleavage through σ-bond metathesis.^48^ Significantly, their DFT studies indicated that in the transition state, there is a strong interaction between the metal and the transferring proton.^49^ A detailed electronic structure analysis confirmed the formation of an M–H bond and the partial oxidation of the metal.^53^

Another example, reported by Lau and co-workers, demonstrated that (Tp)(PPh_3_)Ru(H)(CH_3_CN) (Tp = hydrotris(pyrazolyl)borate) can catalyze H/D exchange between CH_4_ and deuterated organic solvents (e.g., benzene-*d*_6_, tetrahydrofuran-*d*_8_, diethyl ether-*d*_10_, and dioxane-*d*_8_).^50^ The formal oxidation state of Ru is +2 with six electrons in the *d*-orbitals. Using DFT calculations, they found that active (Tp)(PPh_3_)Ru(H) is generated by dissociating CH_3_CN, and this species can break methane C–H bonds to form (Tp)(PPh_3_)Ru(CH_3_)(η^2^-H_2_). Crucially, they found that while the C–H cleavage proceeds through a one-step process reminiscent of σ-bond metathesis, the transition state is characterized by a short bond distance between Ru and the transferring proton (1.57 Å). This outcome suggests that Ru undergoes oxidation during the transition state.^51^

A third example comes from Periana and co-workers, who found that a bis-bidentate O-donor complex, (acac)_2_Ir(CH_3_)(py) (acac = κ^2^ O,O-acetylacetonate, py = pyridine), can catalyze the C–H activation of alkanes (e.g., cyclohexane and *n*-octane) and an arene (e.g., benzene).^52^ The oxidation state of Ir is +3 with six *d*-electrons. The authors proposed that the reaction is initiated by pyridine dissociation followed by acac trans to cis isomerization. Then, the cis-form of (acac)_2_Ir(CH_3_) could activate the alkane or arene C–H bonds through either oxidative addition or σ-bond metathesis. The subsequent DFT studies by Goddard and co-workers revealed that C–H activation occurs via a one-step σ-bond metathesis-like transition state but with a short bond distance between Ir and the transferring proton (1.58 Å).^54^

We calculated the energetics of methane C–H activation by (Cp*)(CO)_2_W(Bcat’) (**2-W**) and (Tp)(PPh_3_)Ru(H) (**2-Ru**). Our results indicate that for **2-W** and **2-Ru**, the reactions indeed proceed via a σ-bond metathesis-like pathway to form (Cp*)(CO)_2_W(CH_3_)(η^2^-H-Bcat’) and (Tp)(PPh_3_)Ru(CH_3_)(η^2^-H_2_), respectively, in a single step. The transition states feature a four-membered ring with short M–H bond lengths (*R*_W–H_ = 1.76 Å for **2-W** and *R*_Ru–H_ = 1.56 Å for **2-Ru**), aligning with the results from previous theoretical studies.^49−51^ The activation and reaction energies (Δ*E*^‡^*/*Δ*E*) are 10.6/0.5 kcal/mol for **2-W** and 9.8/4.8 kcal/mol for **2-Ru**, which are similar to those reported in prior theoretical studies (8.0/0.6 kcal/mol for **2-W**(49) and 13.4/6.9 kcal/mol for **2-Ru**(50)).

**Figure 3:**
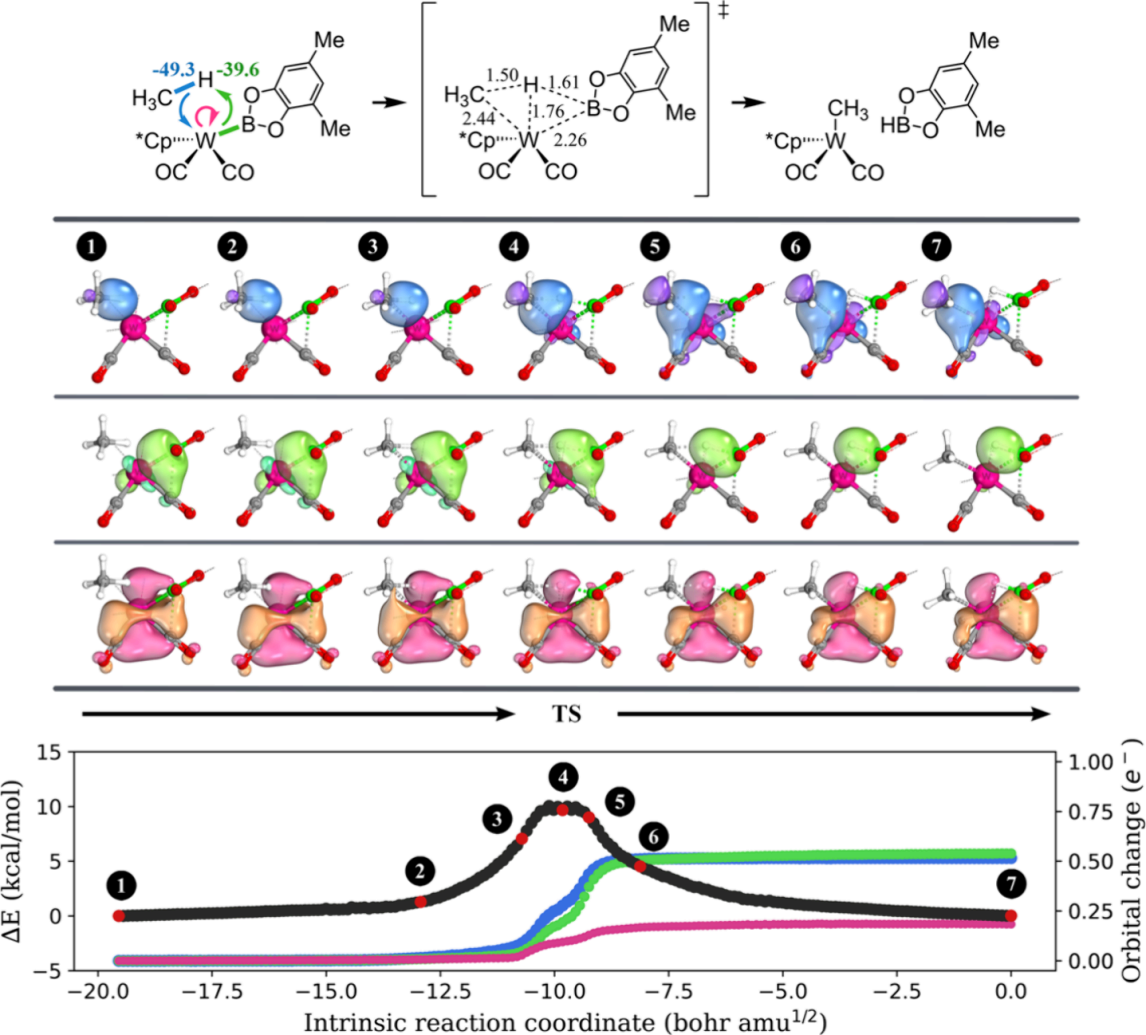
Progression of the IBO localized orbitals throughout the activation of the methane C–H bond through σ-bond metathesis by **2-W**. Each curly arrow is accompanied by a numerical value (in kcal/mol) to indicate its contribution to charge transfer stabilization at the transition state structure, as computed by ALMO-EDA. The important bond lengths in the transition state structure are shown, and the unit is angstrom. For each orbital evolution, a consistent color was used across the three graphs in this figure. A similar analysis was obtained for **2-Ru** + CH_4_ and is summarized in Figure S3 in the SI.

The IBO analysis for **2-W** + CH_4_ and **2-Ru** + CH_4_ reveals an electron flow akin to the nonmetal-assisted σ-bond metathesis, where the methyl group utilizes the electron pair from the cleaved C–H σ-bond to form a new σ-bond with the metal (Figure 3, blue/purple color). Simultaneously, the electron pair in the M-X σ-bond (W–B for **2-W** and Ru–H for **2-Ru**) accommodates the transferring proton, leading to the formation of a new X-H σ-bond (B–H for **2-W** and H–H for **2-Ru**, lime/green color). Crucially, unlike in nonmetal-assisted σ-bond metathesis, our analysis indicates that during this process, a lone pair in the metal’s *d*-orbitals is actively involved in assisting hydrogen migration. This lone pair approaches the transferring proton from the reactant to the TS and then returns to the metal from the TS to the product (pink/orange color). Thus, this reaction mechanism is characterized as [2σ + 2σ, 2*d*-assisted] process.

Interestingly, the IBO plots show some differences between **2-W** and **2-Ru**, even though both are categorized under the same C–H activation mechanism. The potential energy surface and orbital change curves for **2-Ru** are smooth. In contrast, the nonsmooth curves for **2-W** suggest that the reaction may be close to shifting from a one-step to a two-step process. This observation is consistent with previous research, which has demonstrated that one-step metal-mediated σ-bond metathesis can transition to a two-step process—oxidative addition followed by reductive elimination—with slight adjustments to either the ligand or the metal center.^55^

### 3.3 C–H Activation through 1,2-Addition

Organometallic complexes with early transition metals (e.g., Ti, Zr) possessing M = X or M ≡ X multiple bonds (X = C, N, or O) can activate alkane or arene C–H bonds through 1,2-addition. This field of chemistry was pioneered by Wolczanski et al. and Bergman et al. Wolczanski et al. have demonstrated that upon thermolysis, (*t*-Bu_3_SiNH)_3_Zr(CD_3_) is converted to an imido complex, (*t*-Bu_3_SiNH)_2_Zr = NSi-*t*-Bu_3_, accompanied by the extrusion of CD_3_H.^56^ Subsequently, this imido species activates CH_4_, resulting in the formation of (*t*-Bu_3_SiNH)_3_Zr(CH_3_). Bergman et al. have shown that transient (Cp)_2_Zr = N(*t*-Bu), generated through reductive elimination of (Cp)_2_Zr(NH(*t*-Bu))(CH_3_) with extrusion of CH_4_, activates benzene C–H bonds to form (Cp)_2_Zr(NH(*t*-Bu))(Ph).^57^ More recent studies by Mindiola et al. have demonstrated that transient (PNP)Ti≡C-tBu (PNP = N[2-P(CHMe_2_)_2_-4-methylphenyl]_2_), formed through reductive elimination of (PNP)Ti(=CH-tBu)(CH_2_–tBu) with extrusion of CH_3_-tBu, can activate methane and ethane at room temperature to form (PNP)Ti(=CH-tBu)(CH_3_) and (PNP)Ti(=CH-tBu) (C_2_H_5_), respectively.^58,59^

We computed the energetics of methane C–H activation through 1,2-addition by (*t*-Bu_3_SiNH)_2_Zr=NSi-*t*-Bu_3_ (**3-Zr**) and (PNP)Ti≡C-tBu (**3-Ti**). The results from our DFT calculations indicate that in both systems, a methane σ-bond complex is formed with Δ*E* = −11.1 and −12.4 kcal/mol for **3-Zr** and **3-Ti**, respectively. Cundari et al. have studied methane 1,2-addition on (X)_2_M = NH, (M = Ti, Hf, X = H; and M = Zr, X = H, NH_2_, Cl).^60^ They found that stable methane σ-bond complexes can be formed with ∼9.0 kcal/mol stabilization energy, similar to our values. The Δ*E*^‡^/Δ*E*s for C–H cleavage are 16.8/–16.7 and 5.7/–27.6 kcal/mol for **3-Zr** and **3-Ti**, respectively. The computed energetics for **3-Ti** + CH_4_ is consistent with the previous theoretical results reported by Mindiola et al. (Δ*E*^‡^/Δ*E* = 6.9/-26.0 kcal/mol).^58^

**Figure 4:**
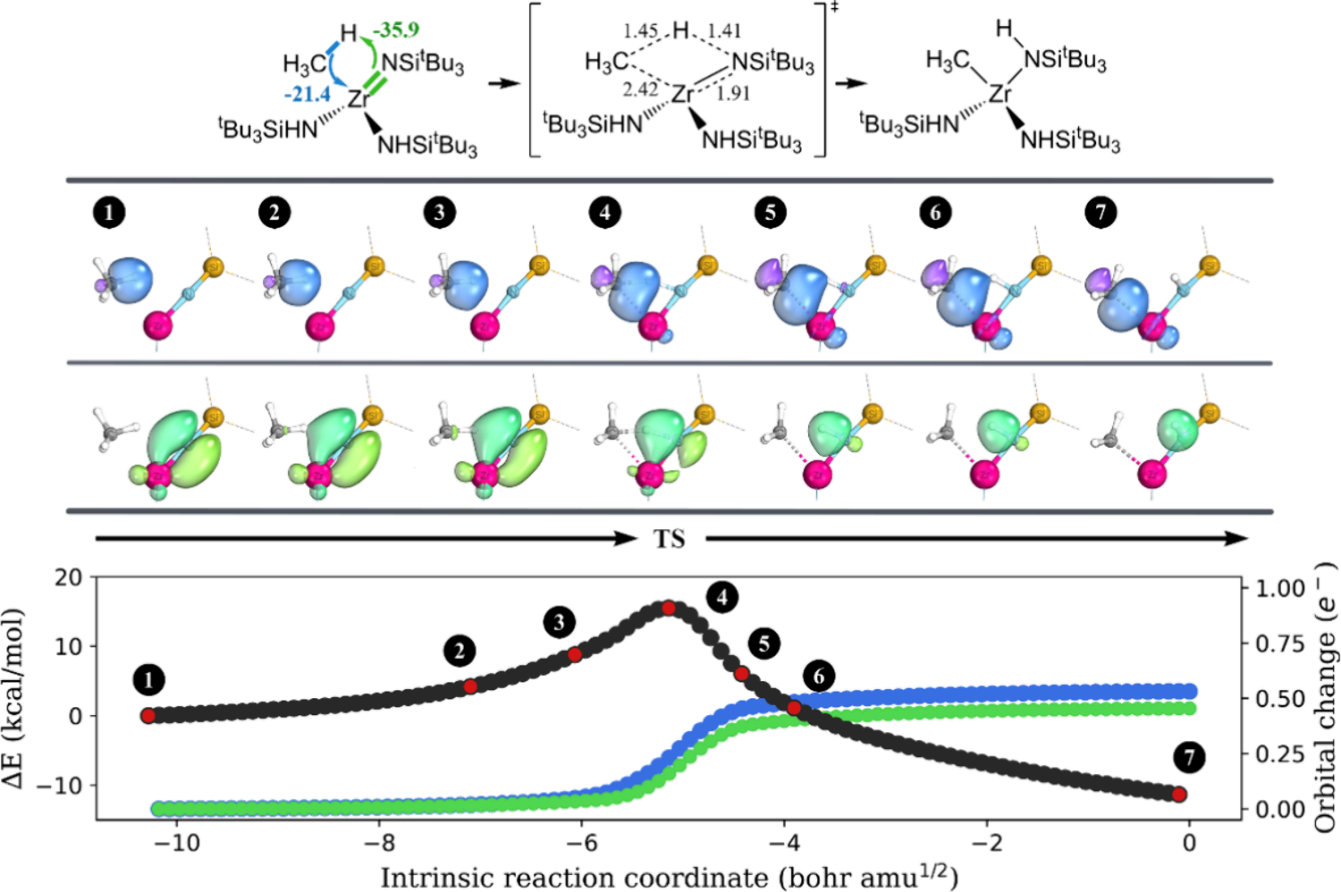
Progression of the IBO localized orbitals throughout the activation of the methane C–H bond through 1,2-addition across Zr=N of **3-Zr**. Each curly arrow is accompanied by a numerical value (in kcal/mol) to indicate its contribution to charge transfer stabilization at the transition state structure, as computed by ALMO-EDA. The important bond lengths in the transition state structure are shown, and the unit is angstrom. For each orbital evolution, a consistent color was used across the three graphs in this figure. A similar analysis was obtained for **3-Ti** + CH_4_ and is summarized in Figure S4 in the SI.

The IBO analysis was performed for two 1,2-addition reactions. We find that similar to oxidative addition and σ-bond metathesis, the methyl group of methane uses the electron pair in the broken C–H bond to form a M–C bond with the metallic center (Figure 4, blue/purple color). In contrast to the aforementioned two C–H activation mechanisms, the electron pair of either the Zr=N or Ti≡C π-orbital is used to host the transferring proton (lime/green color). Thus, this reaction mechanism is characterized as a [2σ + 2π] process.

### 3.4 C–H Activation through Electrophilic Activation

Electrophilic activation involves an electropositive metal that coordinates with and withdraws electron density from C–H bonds. This action increases the acidity of the hydrogen atom, making it more susceptible to abstraction by a lone pair from a heteroatom. The lone pair involved in this process may be provided by an internal or external base. Within the category of internal bases, two distinct types exist, differentiated by the origin of the lone pair, leading to different transition state structures.

#### 3.4.1 Lone Pair on the Metal-bound Heteroatom

The lone pair on the heteroatom that is bound directly to the metallic center (M–X, X = O or N) can be involved in C–H activation. The transition state features a four-membered ring composed of M–X and activated C–H. An example of this C–H activation mechanism is stoichiometric C–H activation reactions of trans-(acac)_2_Ir(OR)(L) (R = H, Me; L = Py, CH_3_OH) with benzene to generate the corresponding phenyl complex with cogeneration of water or methanol, as reported by Goddard and Periana.^8,61^ The DFT calculations showed that after L dissociation, trans-(acac)_2_Ir(OR) transforms to a more active cis-form, which coordinates benzene and activates its C–H bonds. While the original reports suggested that C–H cleavage occurred through σ-bond metathesis,^8,61^ a subsequent study by the same authors revealed the significant role of the lone pair on the oxygen atom of OR in proton abstraction.^9^ This result leads to the recharacterization of the reaction as an internal electrophilic substitution.

Gunnoe and Cundari provided another example of this type of reaction, where TpRu(PMe_3_)_2_(OH) can activate benzene C–H bonds.^62^ This reaction occurs via the exchange of hydrogen from the Ru-bound OH with hydrogen on benzene. According to the DFT calculations by the same authors, after the dissociation of one PMe_3_ ligand, benzene coordinates with Ru. Subsequently, the C–H bond of benzene is activated by transferring a proton to the Ru-bound OH, resulting in the formation of transient TpRu(PMe_3_)(Ph)(H_2_O). The lone pair on the Ru-bound OH was proposed to play a crucial role in accommodating the transferring proton.^63^

We employed DFT calculations to evaluate the energetics of using cis-(acac)_2_Ir(OH) (**4-Ir**) and TpRu(PMe_3_)(OH) (**4-Ru**) to activate methane. Stable methane complexes are formed prior to C–H cleavage with Δ*E*s of only −2.7 and −5.0 kcal/mol for **4-Ir** and **4-Ru**, respectively, indicating weak coordination. The Δ*E*^‡^/Δ*E*s for C–H cleavage are 9.9/–17.9 and 15.4/3.1 kcal/mol for **4-Ir** and **4-Ru**, respectively. The barriers calculated for **4-Ir** and **4-Ru** align with previous theoretical studies reported by Ess et al. (10.4 and 14.8 kcal/mol, respectively).^64^

**Figure 5:**
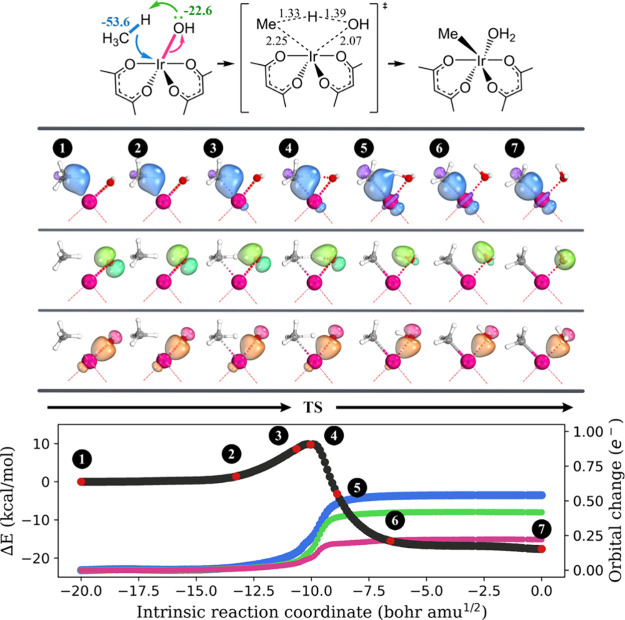
Progression of the IBO localized orbitals throughout the activation of the methane C–H bond through electrophilic activation by **4-Ir**. Each curly arrow is accompanied by a numerical value (in kcal/mol) to indicate its contribution to charge transfer stabilization at the transition state structure, as computed by ALMO-EDA. The important bond lengths in the transition state structure are shown, and the unit is angstrom. For each orbital evolution, a consistent color was used across the three graphs in this figure. A similar analysis was obtained for **4-Ru** + CH_4_ and is summarized in Figure S5 in the SI.

The IBO analysis was conducted on the two reactions. Our findings reveal that akin to oxidative addition, σ-bond metathesis, and 1,2-addition, the methyl group in methane utilizes the electron pair in the broken C–H bond to form a σ-bond with the metal center (Figure 5, blue/purple color). The lone pair electron on the metal-bound OH group is indeed used to accommodate the transferring proton (lime/green color). Moreover, our analysis also indicates that during the reaction, the M–OH covalent bond transitions into a M→OH dative bond (pink/orange color). Consequently, the oxidation state of the metal center remains unchanged throughout the reaction.

#### 3.4.2 Lone Pair on the Pendent Heteroatom

The lone pair accommodating the transferring proton can reside on a pendent heteroatom that is not directly bound to the metal. A well-known example of this is the lone pair on the pendent oxygen atom of a carboxylate ligand. In this scenario, C–H cleavage proceeds through a six-membered ring transition state. This unique pathway, initially proposed in the 1980s,^65^ was first verified by Davies et al.^66^ and Fagnou et al.^67,68^ nearly two decades ago. Davies et al. used DFT calculations to investigate the internal arene C–H activation in Pd(OAc)_2_(dimethylbenzylamine).^66^ They found that C–H activation occurs via deprotonation by the oxygen of the bound acetate, involving a six-membered ring transition state. Fagnou et al. showed that Pd(OAc)_2_ is capable of activating both intermolecular arene C–H bonds^67^ and intramolecular sp^3^ C–H bonds.^68^ Their DFT calculations indicated that C–H cleavage also proceeds via a six-membered ring transition state, involving the transfer of the proton to the pendent oxygen atom of the metal-bound acetate.

It is important to recognize that in addition to the pendent oxygen, the oxygen atom used by the carboxylate to bind with the metal can participate in proton abstraction from C–H bonds through a four-membered ring transition state. However, previous research indicates that this pathway is less favorable compared to the six-membered ring mechanism, which is due to the higher energy requirement needed to distort the structure to form the four-membered ring transition state.^69^

This unique mechanism can also be used to activate alkane C–H bonds. Periana et al. found that (NNC)Ir (NNC = η^3^-6-phenyl-2,2′-bipyridine) can catalytically activate the C–H bond of methane in trifluoroacetic acid.^70^ Nishiyama et al. found that (phebox)Ir(OAc)_2_(H_2_O) (phebox = bis(oxazolinyl)phenyl) can activate alkane C–H bonds (i.e., *n*-heptane and *n*-octane) to form the corresponding (phebox)Ir(OAc)(alkyl).^71^ DFT calculations showed that in both systems, C–H cleavage occurs by transferring the proton from the activated C–H bond to the pendent oxygen of the acetate ligand, proceeding through a six-membered ring transition state.^70,72^

We used DFT calculations to compute the energetics of methane C–H activation by (NNC)Ir(TFA)_2_ (**4-Ir**_**NNC**_, TFA = trifluoroacetate) and (phebox)Ir(OAc)_2_ (**4-Ir**_**phebox**_). In both systems, methane approaches and reacts with Ir from the axial direction. This choice is based on previous research indicating that such a configuration results in a lower kinetic barrier.^70,72^ We find that although methane can form a σ-bond complex with both Ir complexes, the Δ*E*s are 3.6 and 13.4 kcal/mol for **4-Ir**_**NNC**_ and **4-Ir**_**phebox**_, respectively. The formation of the methane σ-bond complex is characterized as endothermic in contrast to the observed exothermic nature in the previously mentioned systems. This outcome can be ascribed to the energy required for TFA or OAc to transition from a bidentate to a monodentate ligand, a necessary step for generating a vacant site for methane coordination. For the subsequent C–H cleavage, Δ*E*^‡^/Δ*E*s are 13.6/2.9 and 7.3/-6.1 kcal/mol for **4-Ir**_**NNC**_ and **4-Ir**_**phebox**_, respectively. The energetics for **4-Ir**_**phebox**_ are similar to the number reported by Cundari et al. (Δ*G*^‡^ = 3.9 kcal/mol).^72^

**Figure 6:**
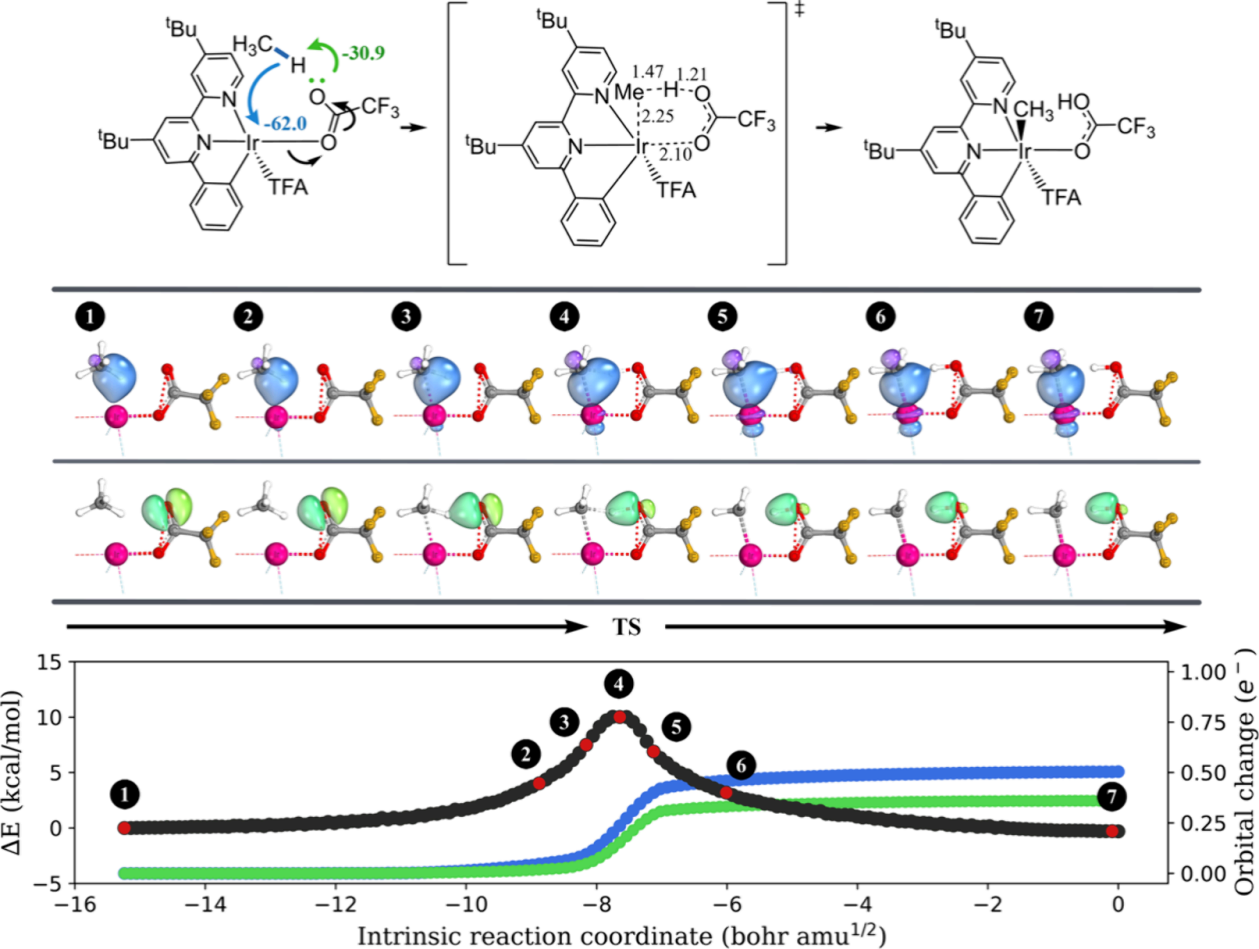
Progression of the IBO localized orbitals throughout the activation of the methane C–H bond through electrophilic activation by **4-Ir**_**NNC**_ via a six-membered ring transition state. Each curly arrow is accompanied by a numerical value (in kcal/mol) to indicate its contribution to charge transfer stabilization at the transition state structure, as computed by ALMO-EDA. The important bond lengths in the transition state structure are shown, and the unit is angstrom. For each orbital evolution, a consistent color was used across the three graphs in this figure. A similar analysis was obtained for **4-Ir**_**phebox**_ + CH_4_ and is summarized in Figure S6 in the SI.

The IBO analysis was performed for the two reactions. We find that similar to the three aforementioned C–H activation mechanisms, the methyl group uses the electron pair in the broken C–H bond to form a M–C bond (Figure 6, blue/purple color). The electron pair on the pendent carboxylate oxygen rather than that from the C=O π-orbital is used to host the transferring proton (lime/green color). In combination with the discussion from the previous section, we determined that electrophilic activation involves a [2σ + 2n] process. The IBO analysis yielded consistent results across all six types of C–H activation mechanisms when ethane, rather than methane, was used as the representative alkane (Figures S8 to S13). This suggests that the electron dynamics observed in methane C–H activation applies to other alkanes as well.

### 3.5 Identification of Primary Orbital Interactions using ALMO-EDA

In this section, we employed ALMO-EDA to analyze the transition states. This EDA scheme breaks down the interaction between metal complexes and methane into distinct and physically insightful components including dispersion, electrostatics, polarization, and charge transfer terms.^26^ Previously, Ess, Periana, and Goddard employed this approach to study C–H activation, and they also explored the nature of charge transfer between metal complexes and activated methane to classify the reactions as electrophilic, ambiphilic, or nucleophilic.^73,74^ Similarly, we have adopted this method to investigate charge transfer between N-Heterocyclic carbenes and gold surfaces.^75^ In our current study, we leverage the EDA framework to pinpoint the complementary occupied/virtual pairings (COVPs) that play a pivotal role in the charge transfer that stabilizes the transition states.

**Figure 7:**
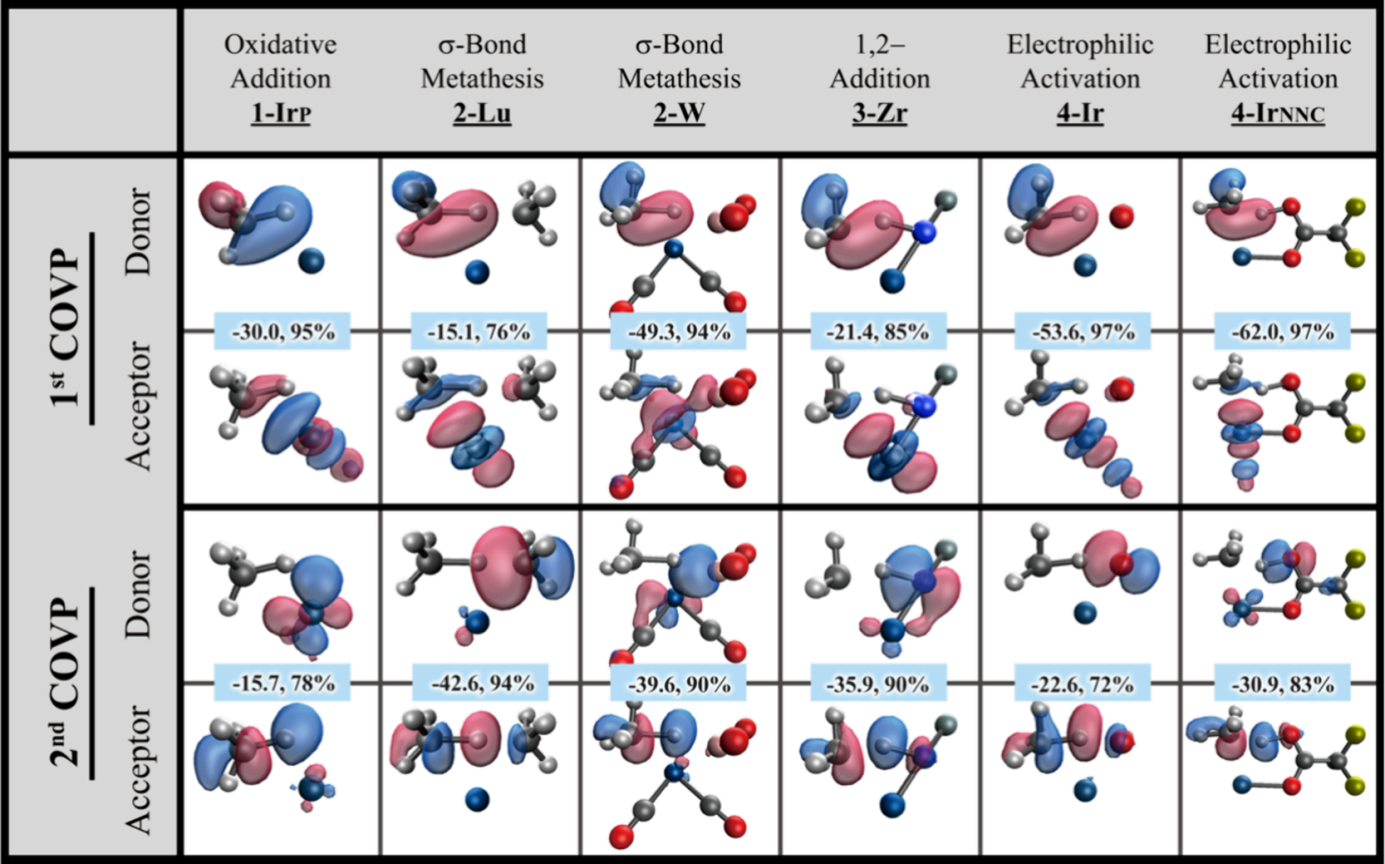
Complementary occupied/virtual pairs (COVPs) in the transition states of methane C–H activation. Only the COVPs that make a major contribution to the charge transfer are shown. Some parts of the metal complexes are omitted for clarity. Each COVP is accompanied by two numbers. The first one indicates the magnitude of stabilization provided by this COVP (in kcal/mol), and the second one is the percentage of this COVP relative to the total charge transfer stabilization for either metal complex → CH_4_ or metal complex ← CH_4_. The analysis for the other six transition states is provided in Figure S7 in the SI.

Our findings indicate that each C–H activation reaction features two dominant COVPs, as illustrated in Figure 7. The first COVP, consistent across all mechanisms, involves the transfer of electron density from the occupied C–H σ-bond to an unoccupied metal *d*-orbital. However, the second COVP varies among the four mechanisms and involves electron transfer to an unoccupied C–H σ*-bond from different sources: a metal *d*-orbital (oxidative addition), an M-C σ-orbital (σ-bond metathesis), an M=N or M≡C π-orbital (1,2-addition), or a lone pair on a metal-bound OH or a pendant oxygen on a carboxylate (electrophilic activation). Thus, the charge transfer (or electron flow) revealed by COVPs aligns with the insights from the IBO analysis, which demonstrates that in all four C–H activation mechanisms, the methyl group in methane uses the electron pair from the cleaved C–H bond to form a σ-bond with the metal while the electron pair facilitating proton transfer varies with each mechanism.

Moreover, ALMO-EDA facilitates the quantification of charge transfer stabilization attributed to each COVP. This capability enables us to assess the contributions of the two primary COVPs within each mechanism. For example, for the methane oxidative addition on **1-Ir**_**P**_, electron transfer from the C–H σ-bond to a Ir *d*-orbital (−30.0 kcal/mol) proves to be more significant than the transfer from another Ir *d*-orbital to the C–H σ*-bond (−15.7 kcal/mol). For methane σ-bond metathesis on **2-Lu**, the electron transfer from the C–H σ-bond to a Lu *d*-orbital (−15.1 kcal/mol) is less pronounced compared to the transfer from the Lu–C σ-orbital to the C–H σ*-bond (−42.6 kcal/mol).

## 4 Conclusions

In this study, we revisited representative reactions of four alkane C–H activation mechanisms using DFT calculations combined with IBO analysis. This approach allowed for visualization of electron flow during the reactions and yielded a clear characterization of the different C–H activation mechanisms. We observed that in all four mechanisms, the methyl group of methane utilizes the electron pair from the broken C–H bond to form a σ-bond with the metal. However, the specific electron pair accommodating the transferring proton varies across the mechanisms: in oxidative addition, it originates from d-orbitals; in σ-bond metathesis, it arises from the M-X σ-bond; in 1,2-addition, it comes from the π-bond of M-X multiple bonds; and in electrophilic activation, it originates from lone pairs on heteroatoms. This insight provides valuable guidance for tunning catalyst reactivity*.* The electron dynamics revealed through the IBO analysis are consistent with the findings from the ALMO-EDA, which additionally quantifies the two primary interactions in each process. These insights deepen our understanding of the electron movement involved in alkane C–H activation, significantly advancing our knowledge of these pivotal reactions.

## Supporting Information Available

Optimized coordinates as well as some additional tables and figures (PDF)
CH activation IBO structures (ZIP)
