# Supplementary material for: Electron Dynamics in Alkane C–H Activation Mediated by Transition Metal Complexes
Source: J Phys Chem A. 2024 Jun 4;128(23):4638–50. doi: 10.1021/acs.jpca.4c01131 (PMC11182348; doi:10.1021/acs.jpca.4c01131)
Supplement: Supplementary file 1 — jp4c01131_si_001.pdf [file jp4c01131_si_001.pdf]

Supporting Information for

Electron Dynamics in Alkane C-H Activation Mediated by Transition Metal Complexes

Yu-Ho Cheng,† Yeu-Shiuan Ho,† Chia-Jung Yang, Chun-Yu Chen, Chi-Tien Hsieh, and Mu-Jeng Cheng*

Department of Chemistry, National Cheng Kung University, Tainan, 701, Taiwan

Re-Submitted to
J. Phys. Chem. A
May 15th, 2024

Corresponding Author
*Email: mjcheng@mail.ncku.edu.tw

Table S1. Energetics of methane C-H activation across different metal complexes computed using the B3LYP-D3/def2-TZVP//B3LYP-D3/def2-SVP level of theory. All energetics are referenced to isolated metal complex and methane.

Metal Complex	Methane Complex	TS	Product
	**Oxidative Addition**		
$(Cp^*)(PMe_3)Ir$ (**1-Ir$_P$**)	-15.6	-17.5	-46.2
$(Cp^*)(CO)Ir$ (**1-Ir$_C$**)	-14.8	-18.6	-37.8
	**σ-Bond Metathesis**		
$(Cp^*)_2Lu(CH_3)$ (**2-Lu**)	-4.6	15.9	-4.6
$(Cp^*)_2Sc(CH_3)$ (**2-Sc**)	-2.2	16.9	-2.2
$(Cp^*)(CO)_2W(Bcat')$ (**2-W**)	-8.2	1.8	-8.3
$(Tp)(PPh_3)Ru(H)$ (**2-Ru**)	-7.4	3.2	-2.4
	**1,2-Addition**		
$(t\text{-}Bu_3SiNH)_2Zr{=}NSi\text{-}t\text{-}Bu_3$ (**3-Zr**)	-9.8	8.5	-25.2
(PNP)Ti≡C-tBu (**3-Ti**)	-10.3	-3.9	-37.7
	**Electrophilic Activation**		
$cis\text{-}(acac)_2Ir(OH)$ (**4-Ir**)	-1.3	10.1	-16.0
$TpRu(PMe_3)(OH)$ (**4-Ru**)	-2.8	14.8	2.0
(NNC)Ir(III)(TFA) (**4-Ir$_{NNC}$**)	5.9	19.7	9.4
$(phebox)Ir(OAc)_2$ (**4-Ir$_{Phebox}$**)	14.6	23.1	10.0

Discussion of Orbital Changes

Analyzing the orbital changes (OCs) of the two primary IBO evolutions in each reaction, we found that the OCs associated with metal-carbon bond formation are relatively consistent across all six reactions (0.55 ~ 0.50 e^-, Table S2). However, the OCs for proton transfer vary significantly (0.71 ~ 0.34 e^-), as each reaction utilizes a different molecular orbital to accommodate the proton, resulting in varied charge distributions. Interestingly, the OC for proton transfer correlates with the charge transferred to the proton. The **1-Ir$_P$** + CH_4 reaction exhibits the largest OC (0.71 e^-), involving the most significant charge transfer to the proton (from -0.07 to -1.10 e^-). Conversely,

4-Ir + CH_4 and **4-Ir$_{NNC}$** + CH_4 show the smallest OCs (0.42 and 0.34 e$^-$, respectively), with the least charge transfer to the proton (from 0.00 to -0.61 e$^-$ and from 0.00 to -0.56 e$^-$, respectively). This results in a larger OC discrepancy between the two primary IBO evolutions in **1-Ir$_P$** + CH_4, **4-Ir** + CH_4, and **4-Ir$_{NNC}$** + CH_4.

Table S2. Orbital changes (OCs) and corresponding charge distributions of the two most significant IBO evolutions in the six methane C-H activation reactions.

Species	M-C Formation			Proton Transfer			|ΔOC|
	OC	Reactant Charge Distribution	Product Charge Distribution	OC	Reactant Charge Distribution	Product Charge Distribution	
**1-Ir$_P$**	0.52	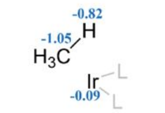	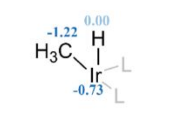	0.71	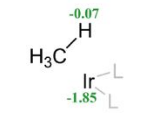	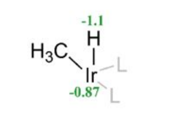	0.19
**2-Lu**	0.55	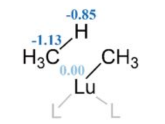	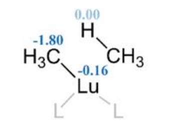	0.55	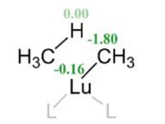	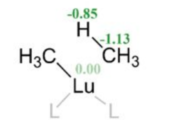	0.00
**2-W**	0.52	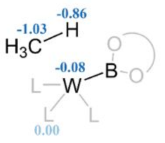	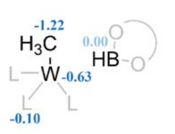	0.54	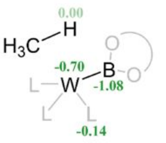	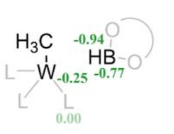	0.02
**3-Zr**	0.53	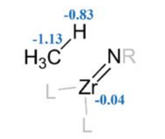	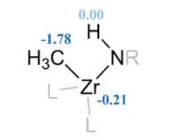	0.45	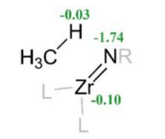	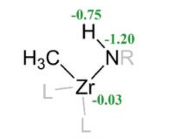	0.08
**4-Ir**	0.54	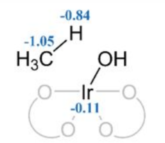	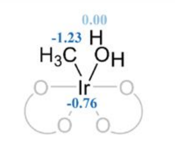	0.42	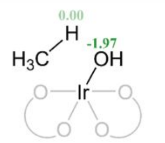	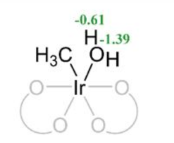	0.12
**4-Ir$_{NNC}$**	0.50	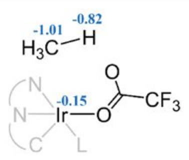	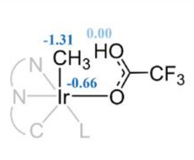	0.34	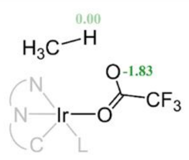	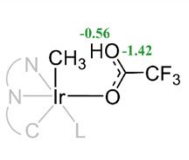	0.16

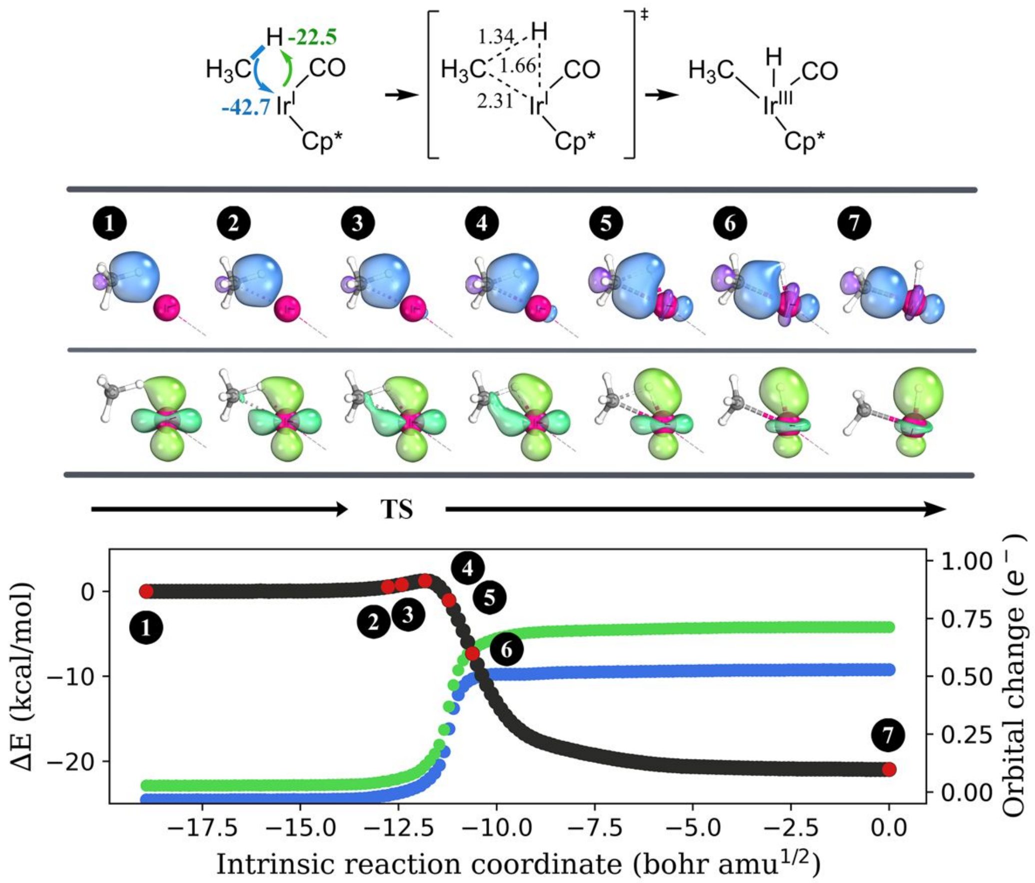

Figure S1. Progression of the IBO localized orbitals throughout methane oxidative addition on **1-Ir$_{\mathbf{C}}$**. Each curly arrow is accompanied by a numerical value (in kcal/mol) to indicate its contribution to charge transfer stabilization at the transition state structure, as computed by ALMO-EDA. The important bond lengths in the transition state structure are shown, and the unit is angstrom. For each orbital evolution, a consistent color was used across the three graphs in this figure.

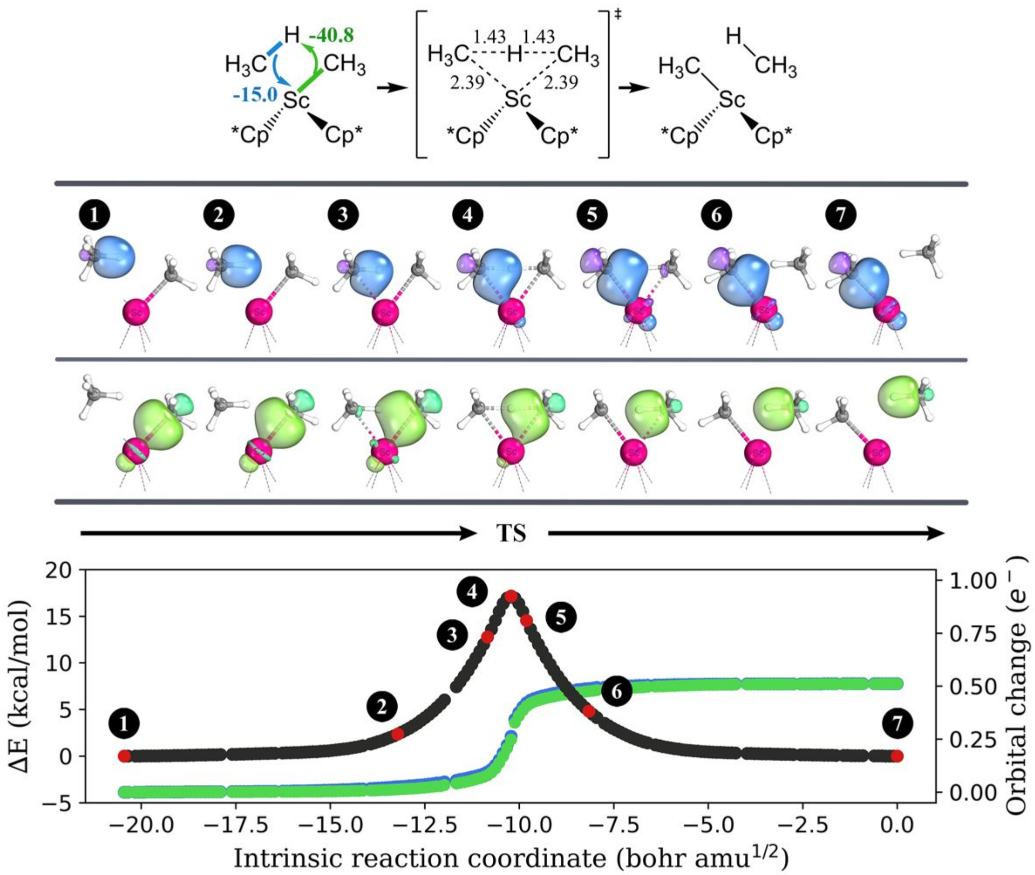

Figure S2. Progression of the IBO localized orbitals throughout the activation of the methane C-H bond via σ-bond metathesis by **2-Sc**. Each curly arrow is accompanied by a numerical value (in kcal/mol) to indicate its contribution to charge transfer stabilization at the transition state structure, as computed by ALMO-EDA. The important bond lengths in the transition state structure are shown, and the unit is angstrom. For each orbital evolution, a consistent color was used across the three graphs in this figure.

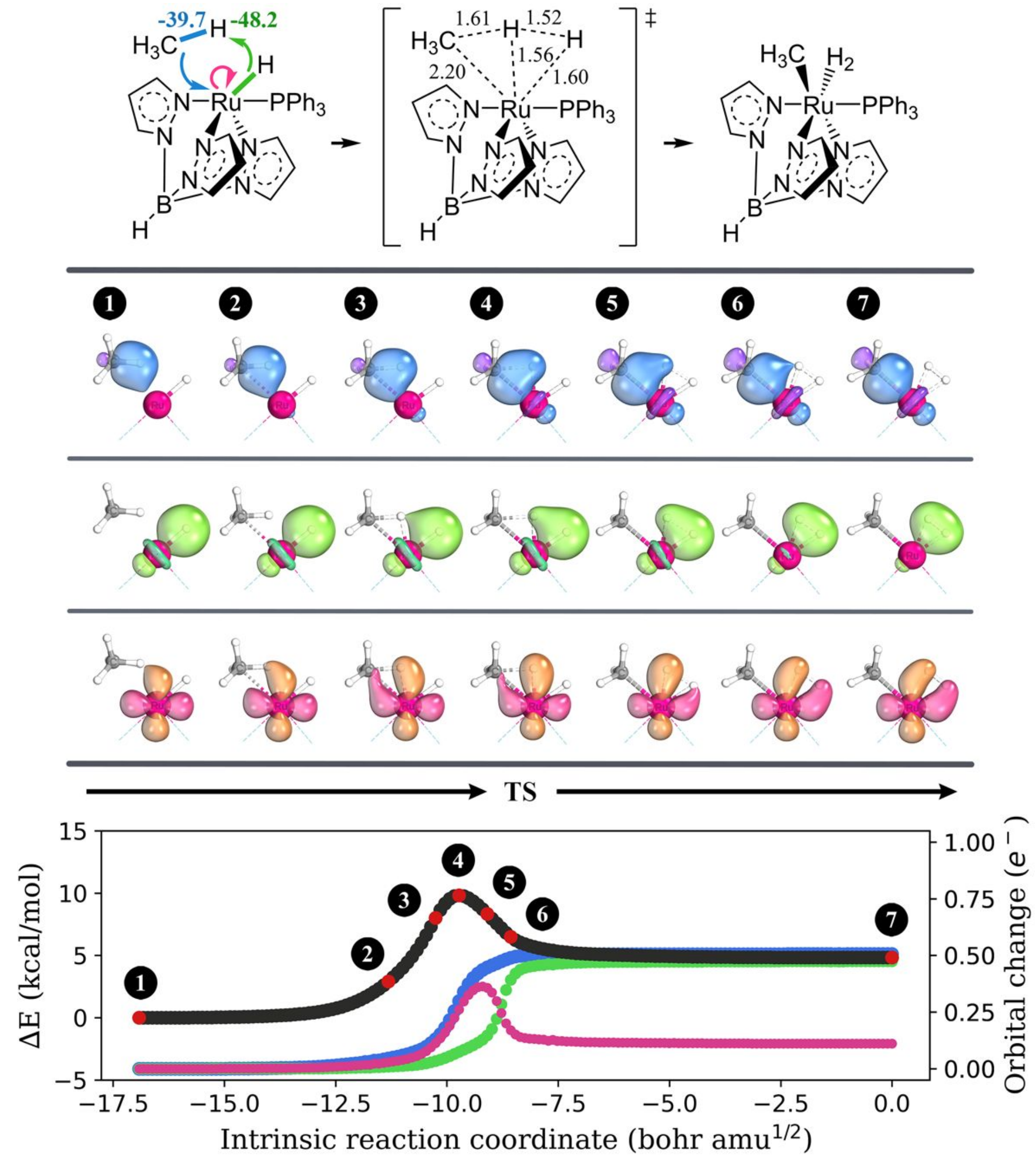

Figure S3. Progression of the IBO localized orbitals throughout the activation of the methane C-H bond through σ-bond metathesis by **2-Ru**. Each curly arrow is accompanied by a numerical value (in kcal/mol) to indicate its contribution to charge transfer stabilization at the transition state structure, as computed by ALMO-EDA. The important bond lengths in the transition state structure are shown, and the unit is angstrom. For each orbital evolution, a consistent color was used across the three graphs in this figure.

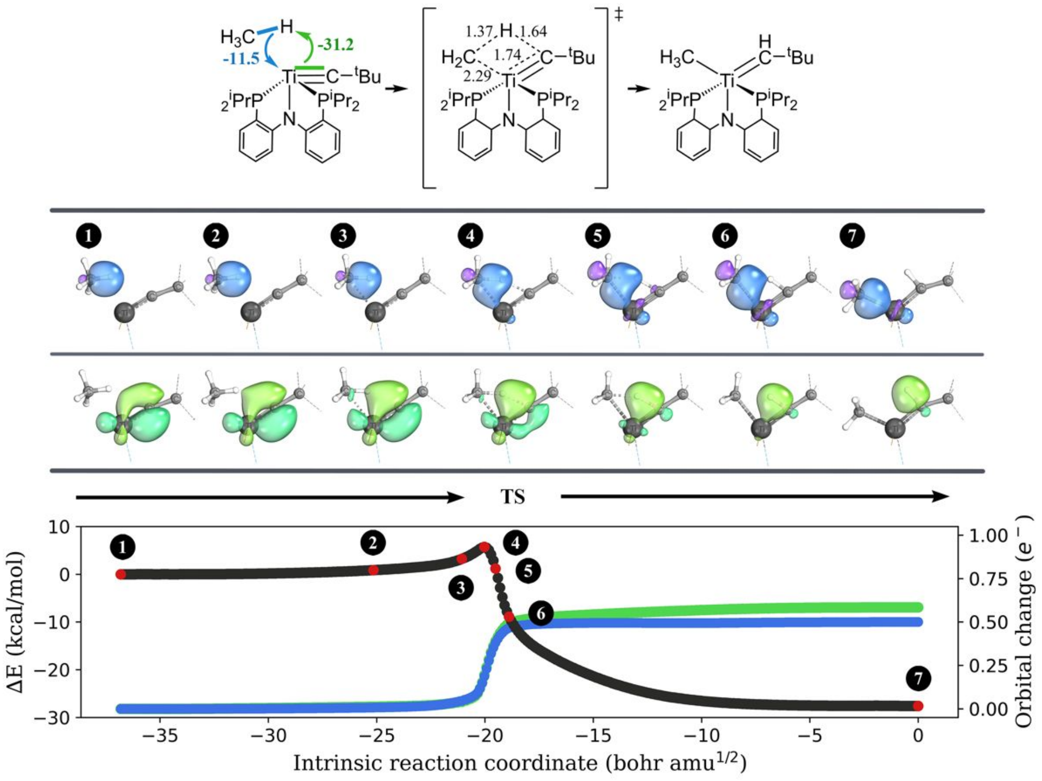

Figure S4. Progression of the IBO localized orbitals throughout the activation of the methane C-H bond through 1,2-addition across Ti ≡ C of **3-Ti**. Each curly arrow is accompanied by a numerical value (in kcal/mol) to indicate its contribution to charge transfer stabilization at the transition state structure, as computed by ALMO-EDA. The important bond lengths in the transition state structure are shown, and the unit is angstrom. For each orbital evolution, a consistent color was used across the three graphs in this figure.

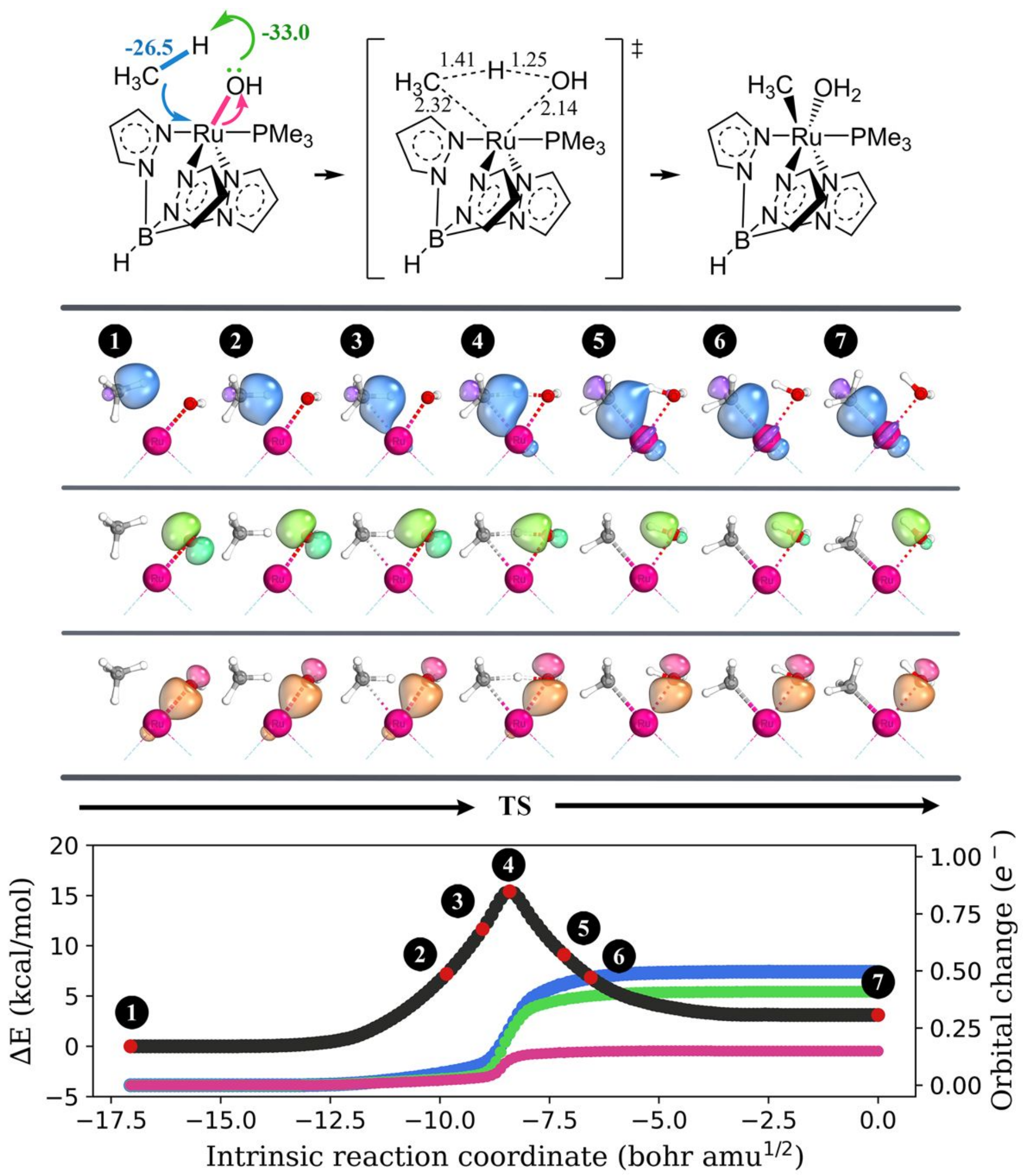

Figure S5. Progression of the IBO localized orbitals throughout the activation of the methane C-H bond through electrophilic activation by **4-Ru**. Each curly arrow is accompanied by a numerical value (in kcal/mol) to indicate its contribution to charge transfer stabilization at the transition state structure, as computed by ALMO-EDA. The important bond lengths in the transition state structure are shown, and the unit is angstrom. For each orbital evolution, a consistent color was used across the three graphs in this figure.

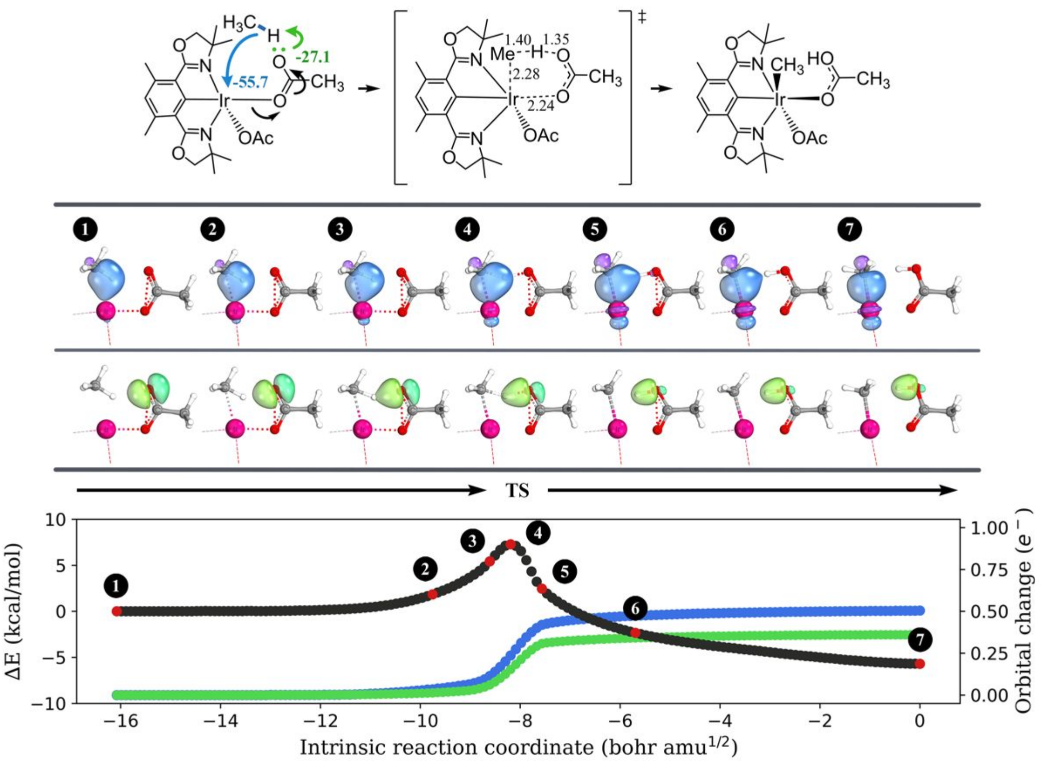

Figure S6. Progression of the IBO localized orbitals throughout the activation of the methane C-H bond through electrophilic activation by **4-Ir$_{\mathbf{phebox}}$** via a six-membered ring transition state. Each curly arrow is accompanied by a numerical value (in kcal/mol) to indicate its contribution to charge transfer stabilization at the transition state structure, as computed by ALMO-EDA. The important bond lengths in the transition state structure are shown, and the unit is angstrom. For each orbital evolution, a consistent color was used across the three graphs in this figure.

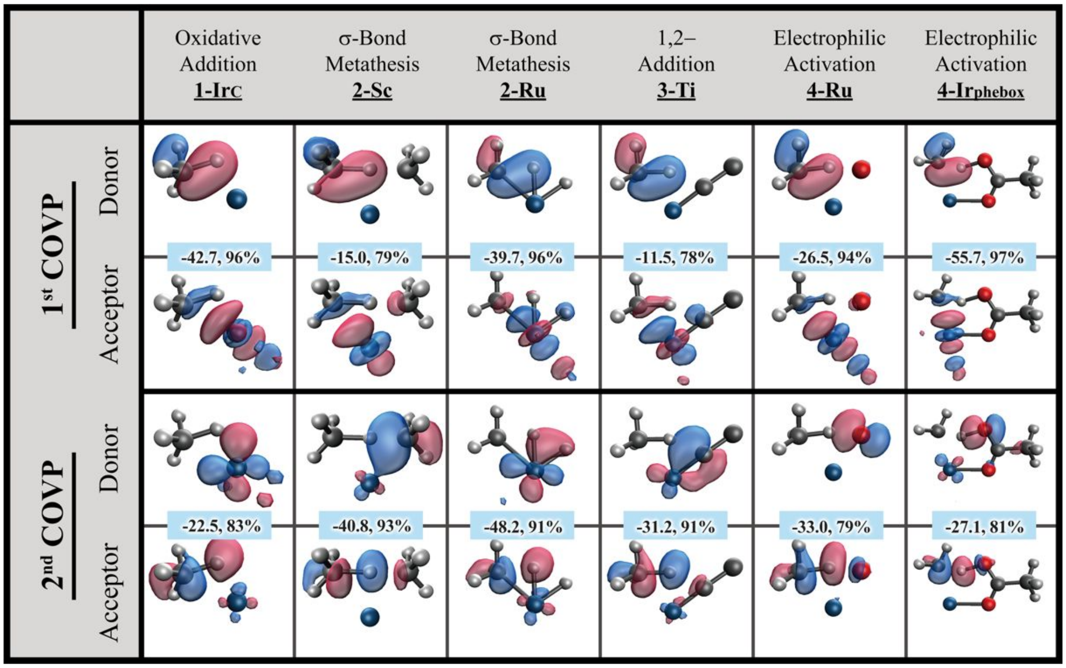

Figure S7. Complementary occupied/virtual pairs (COVPs) in the transition states of methane C-H activation. Only the COVPs that make a major contribution to the charge transfer are shown. Some parts of the metal complexes are omitted for clarity. Each COVP is accompanied by two numbers. The first one indicates the magnitude of stabilization provided by this COVP (in kcal/mol), and the second one is the percentage of this COVP relative to the total charge transfer stabilization for either metal complex → CH_4 or metal complex ← CH_4.

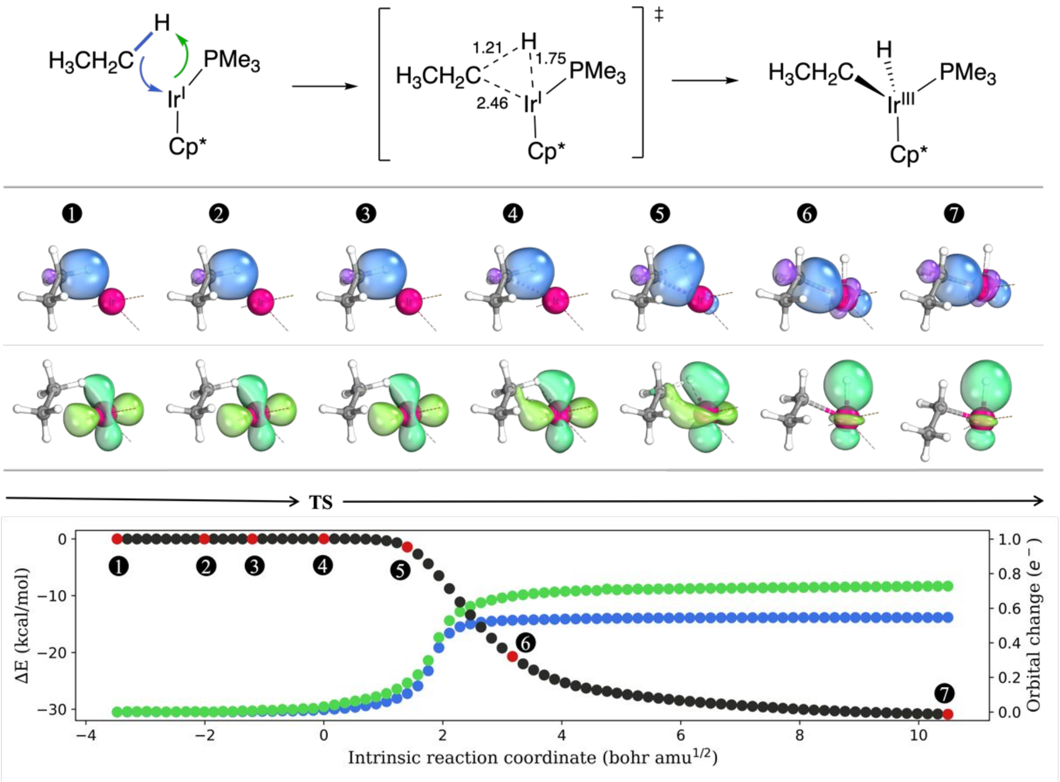

Figure S8. Progression of the IBO localized orbitals throughout ethane oxidative addition on **1-Ir$_P$**. For each orbital evolution, a consistent color was used across the three graphs in this figure. The important bond lengths in the transition state structure are shown, and the unit is angstrom.

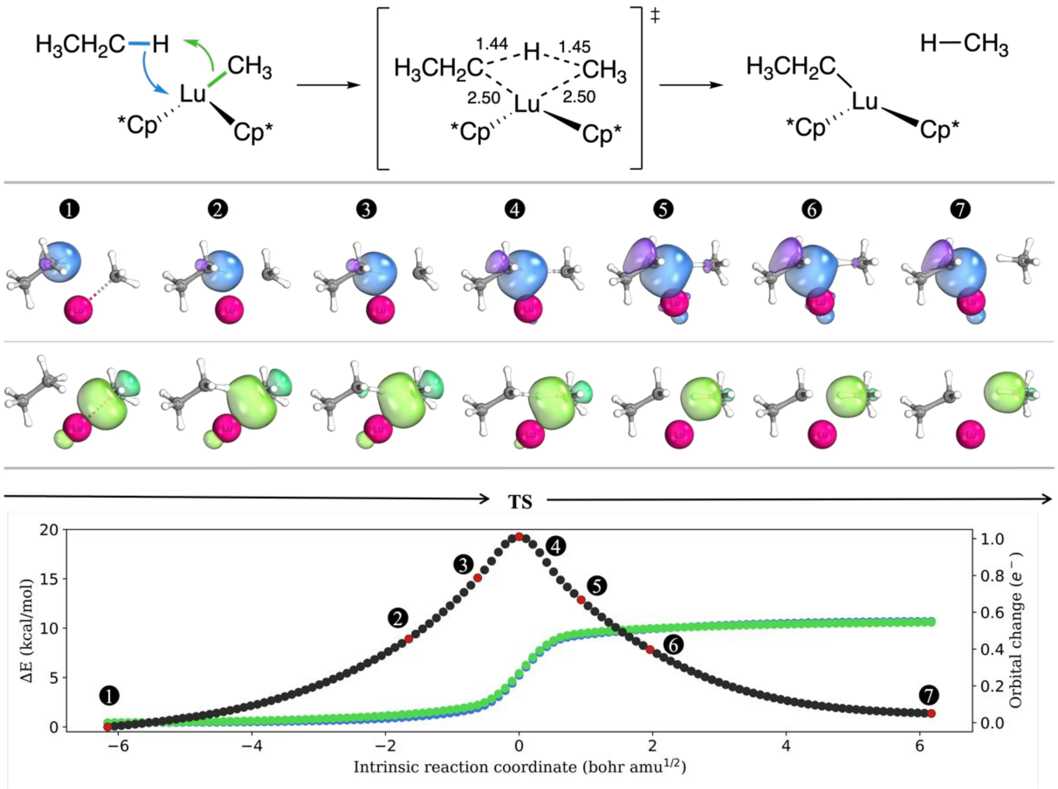

Figure S9. Progression of the IBO localized orbitals throughout the activation of the ethane C-H bond via σ-bond metathesis by **2-Lu**. The important bond lengths in the transition state structure are shown, and the unit is angstrom. For each orbital evolution, a consistent color was used across the three graphs in this figure.

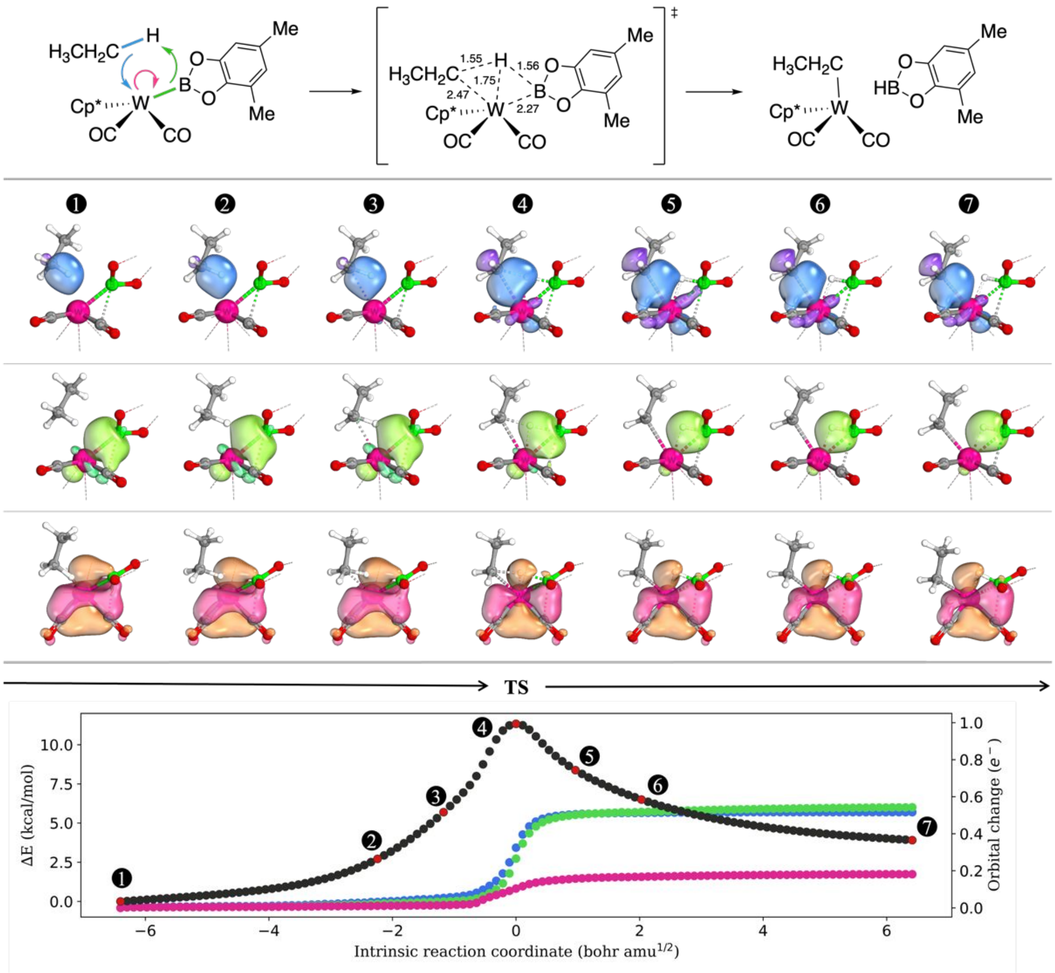

Figure S10. Progression of the IBO localized orbitals throughout the activation of the ethane C-H bond through σ-bond metathesis by **2-W**. The important bond lengths in the transition state structure are shown, and the unit is angstrom. For each orbital evolution, a consistent color was used across the three graphs in this figure.

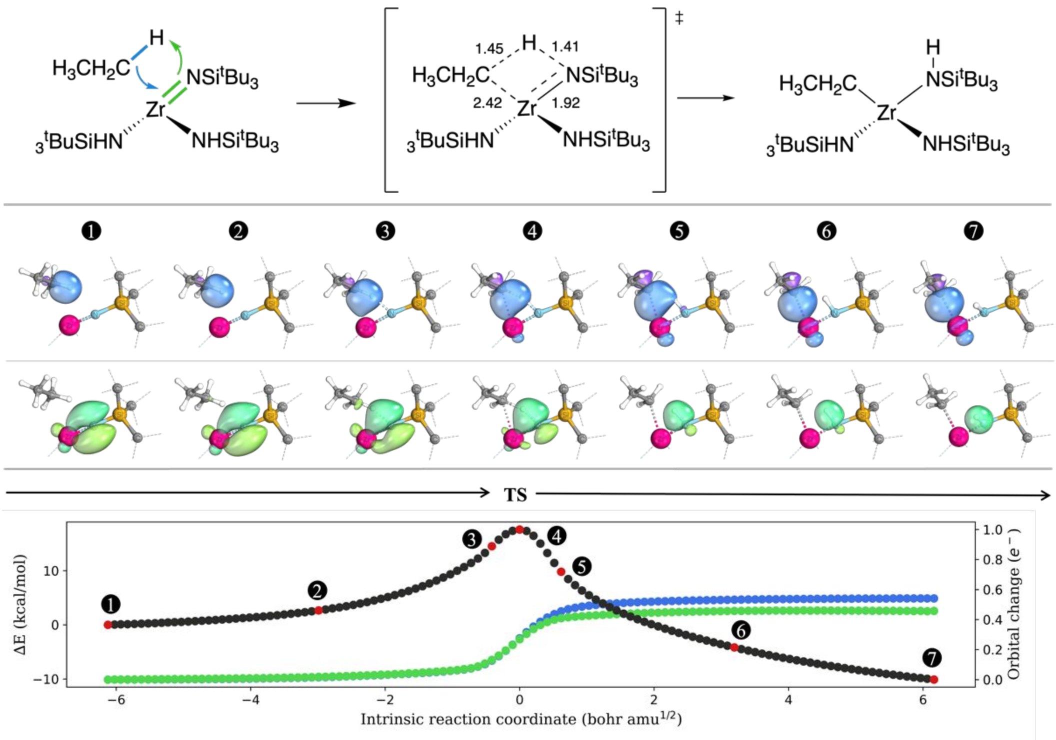

Figure S11. Progression of the IBO localized orbitals throughout the activation of the ethane C-H bond through 1,2-addition across Zr=N of **3-Zr**. The important bond lengths in the transition state structure are shown, and the unit is angstrom. For each orbital evolution, a consistent color was used across the three graphs in this figure.

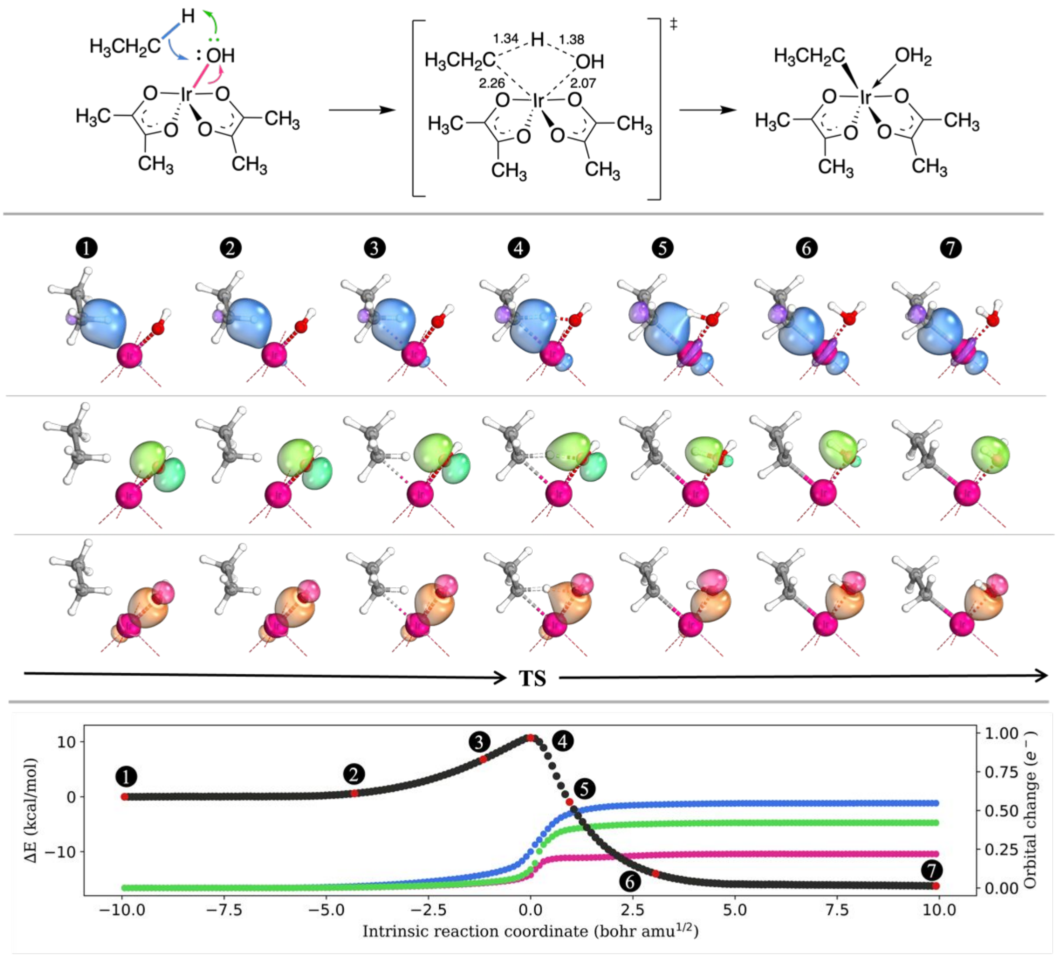

Figure S12. Progression of the IBO localized orbitals throughout the activation of the ethane C-H bond through electrophilic activation by **4-Ir**. The important bond lengths in the transition state structure are shown, and the unit is angstrom. For each orbital evolution, a consistent color was used across the three graphs in this figure.

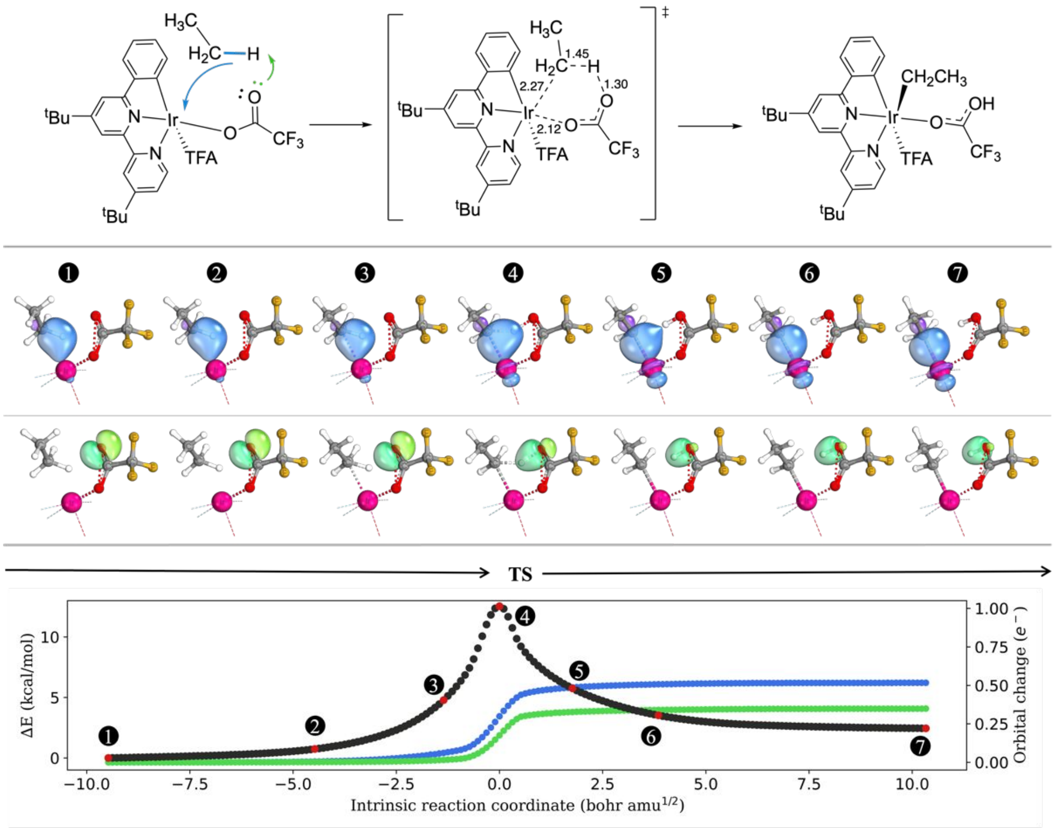

Figure S13. Progression of the IBO localized orbitals throughout the activation of the ethane C-H bond through electrophilic activation by **4-Ir$_{NNC}$** via a six-membered ring transition state. The important bond lengths in the transition state structure are shown, and the unit is angstrom. For each orbital evolution, a consistent color was used across the three graphs in this figure.

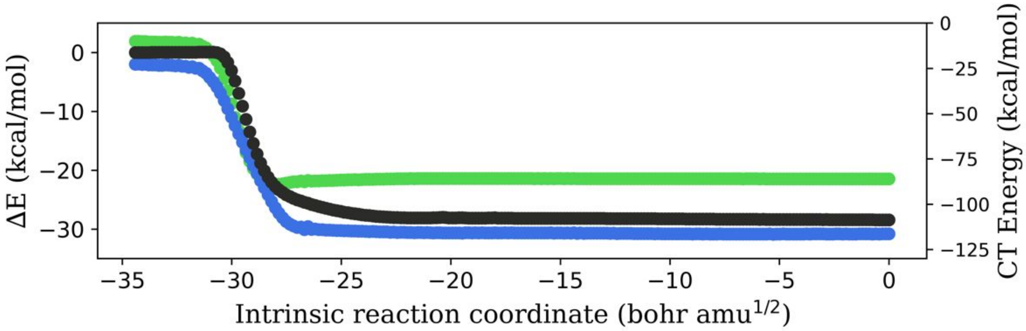

Figure S14. Variations in charge transfer stabilization energies of the two primary COVPs along the IRC of methane C-H activation by **1-Ir$_\mathbf{P}$**. The black line represents the potential energy surface, while the blue and green lines correspond to the COVPs of the "metal complex" → CH_4 and "metal complex" ← CH_4, respectively.

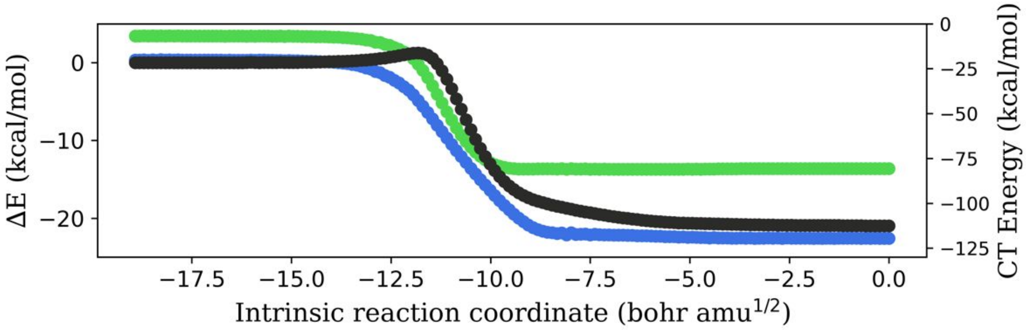

Figure S15. Variations in charge transfer stabilization energies of the two primary COVPs along the IRC of methane C-H activation by **1-Ir$_\mathbf{C}$**. The black line represents the potential energy surface, while the blue and green lines correspond to the COVPs of the "metal complex" → CH_4 and "metal complex" ← CH_4, respectively.

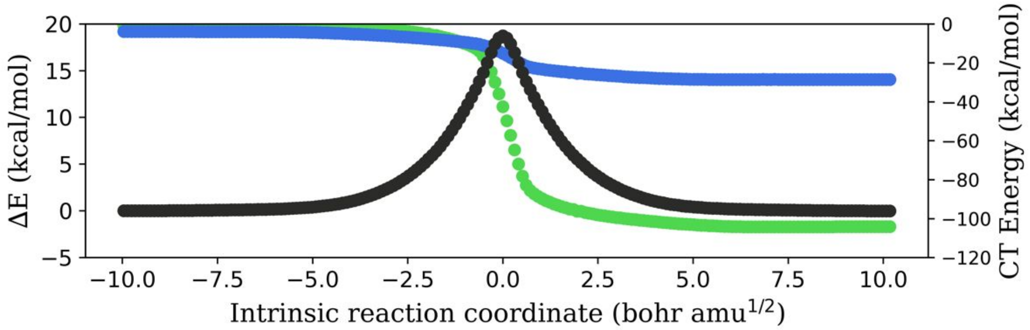

Figure S16. Variations in charge transfer stabilization energies of the two primary COVPs along the IRC of methane C-H activation by **2-Lu**. The black line represents the potential energy surface, while the blue and green lines correspond to the COVPs of the "metal complex" $\rightarrow$ CH_4 and "metal complex" $\leftarrow$ CH_4, respectively.

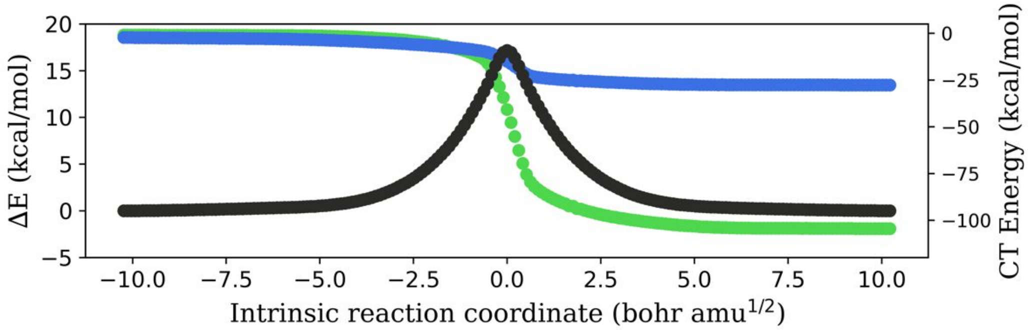

Figure S17. Variations in charge transfer stabilization energies of the two primary COVPs along the IRC of methane C-H activation by **2-Sc**. The black line represents the potential energy surface, while the blue and green lines correspond to the COVPs of the "metal complex" $\rightarrow$ CH_4 and "metal complex" $\leftarrow$ CH_4, respectively.

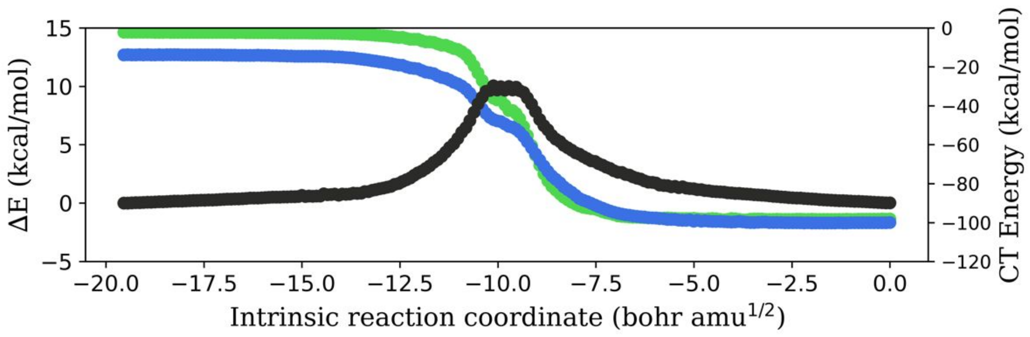

Figure S18. Variations in charge transfer stabilization energies of the two primary COVPs along the IRC of methane C-H activation by **2-W**. The black line represents the potential energy surface, while the blue and green lines correspond to the COVPs of the "metal complex" → CH_4 and "metal complex" ← CH_4, respectively.

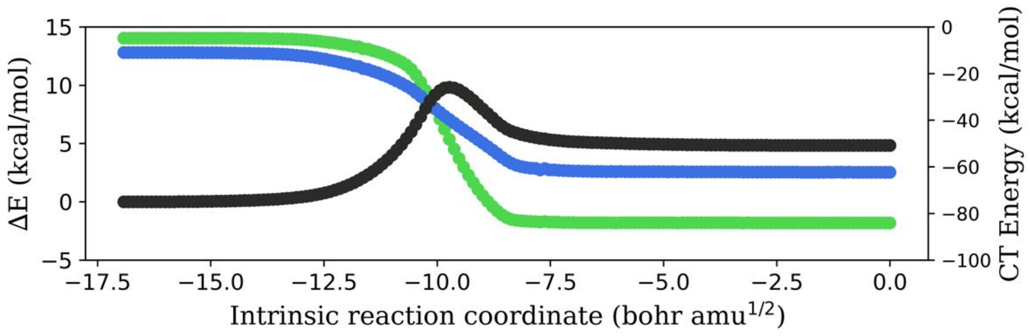

Figure S19. Variations in charge transfer stabilization energies of the two primary COVPs along the IRC of methane C-H activation by **2-Ru**. The black line represents the potential energy surface, while the blue and green lines correspond to the COVPs of the "metal complex" → CH_4 and "metal complex" ← CH_4, respectively.

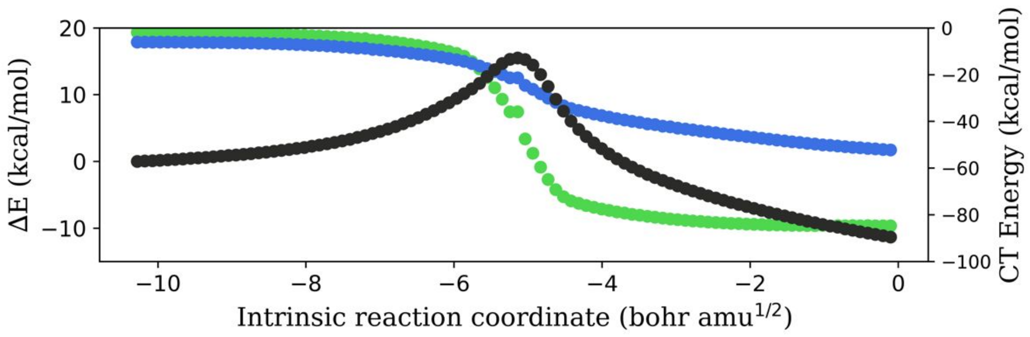

Figure S20. Variations in charge transfer stabilization energies of the two primary COVPs along the IRC of methane C-H activation by **3-Zr**. The black line represents the potential energy surface, while the blue and green lines correspond to the COVPs of the "metal complex" → CH_4 and "metal complex" ← CH_4, respectively.

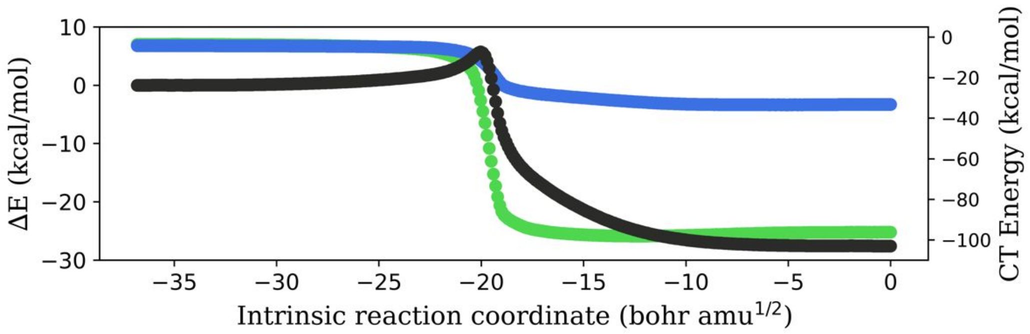

Figure S21. Variations in charge transfer stabilization energies of the two primary COVPs along the IRC of methane C-H activation by **3-Ti**. The black line represents the potential energy surface, while the blue and green lines correspond to the COVPs of the "metal complex" → CH_4 and "metal complex" ← CH_4, respectively.

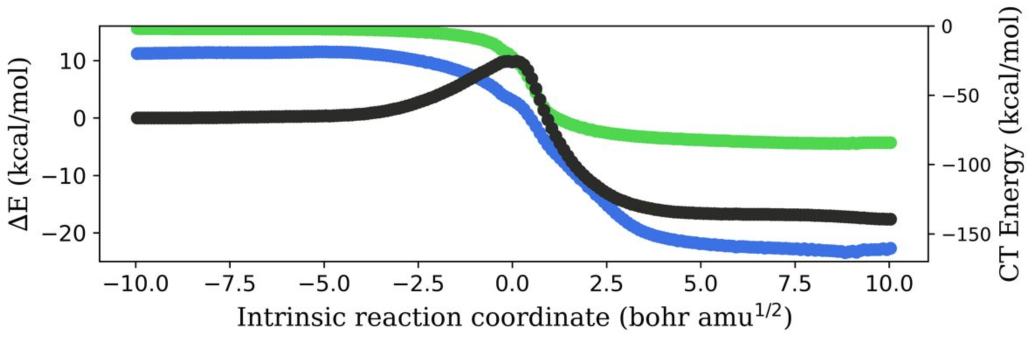

Figure S22. Variations in charge transfer stabilization energies of the two primary COVPs along the IRC of methane C-H activation by **4-Ir**. The black line represents the potential energy surface, while the blue and green lines correspond to the COVPs of the "metal complex" → CH_4 and "metal complex" ← CH_4, respectively.

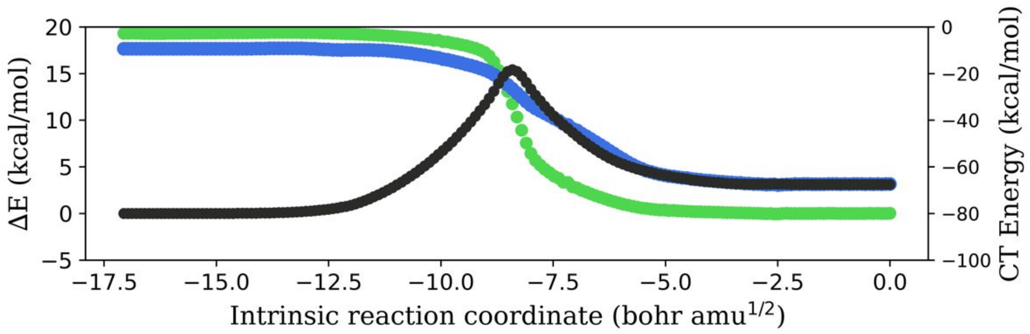

Figure S23. Variations in charge transfer stabilization energies of the two primary COVPs along the IRC of methane C-H activation by **4-Ru**. The black line represents the potential energy surface, while the blue and green lines correspond to the COVPs of the "metal complex" → CH_4 and "metal complex" ← CH_4, respectively.

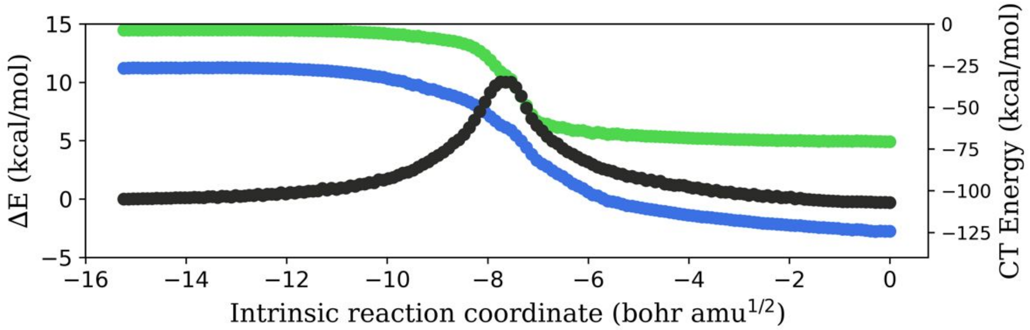

Figure S24. Variations in charge transfer stabilization energies of the two primary COVPs along the IRC of methane C-H activation by **4-Ir$_{\mathbf{NNC}}$**. The black line represents the potential energy surface, while the blue and green lines correspond to the COVPs of the "metal complex" $\rightarrow$ CH_4 and "metal complex" $\leftarrow$ CH_4, respectively.

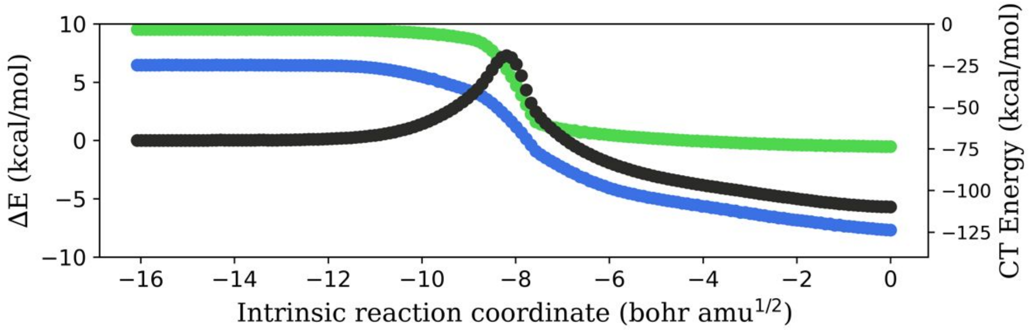

Figure S25. Variations in charge transfer stabilization energies of the two primary COVPs along the IRC of methane C-H activation by **4-Ir$_{\mathbf{Phebox}}$**. The black line represents the potential energy surface, while the blue and green lines correspond to the COVPs of the "metal complex" $\rightarrow$ CH_4 and "metal complex" $\leftarrow$ CH_4, respectively.
